# Co-creation process of an app for people with rare diseases - a citizen science approach

**DOI:** 10.1186/s13023-025-04140-1

**Published:** 2025-11-27

**Authors:** Jannik Schaaf, Michaela Christina Neff, Jörg Scheidt, Holger Storf

**Affiliations:** 1 Institute of Medical Informatics, Goethe University Frankfurt, University Medicine, Theodor-Stern Kai 7, 60590 Frankfurt, Germany; 2 Institute for Information Systems, University of Applied Sciences Hof, Alfons-Goppel-Platz 1, 95028 Hof, Germany

**Keywords:** Citizen science, Mobile health, Rare diseases, Patient involvement

## Abstract

**Background:**

Rare diseases affect a small percentage of the population, leading to challenges such as delayed diagnoses and limited treatment options. Mobile health technologies offer solutions to improve patient outcomes, yet their application in rare diseases remains underexplored. The German citizen science project SelEe created a customizable app for the self-management of rare diseases through a co-creation process that involved patients with such conditions.

**Methods:**

The project consisted of three phases. In Phase 1, 9 to 68 patients or relatives of patients participated in workshops to define research topics and app requirements. Phase 2 involved a core research team of nine patients and researchers who iteratively developed the app, released in March 2023. Phase 3 focused on evaluating the app’s usage and usability through an in-app survey conducted from March 2023 to February 2024. We utilized descriptive statistics to evaluate app usage and employed the mHealth App Usability Questionnaire to assess usability.

**Results:**

The SelEe app offers the possibility to create and store data in a personalized health diary. Patients can create their own templates or use templates which were defined by the core research team. Users can record findings (e.g. blood test results) and export data using different graphs and formats. Furthermore, the app supports blind users. The app was downloaded 3040 times and 1456 users registered, with 1967 unique diseases entered. 50.7% of the diseases were rare, 30.5% non-rare, and 18.8% were classified as suspected, undefined, or symptoms. A total of 1223 valid user profiles were analyzed for app usage and demographics. Furthermore, 432 users qualified for the in-app survey by making at least one health diary entry, and 117 participated. The app was rated with an overall usability score of 5.19 out of 7. While the app’s health diary function was frequently used, other functionalities like findings and data export were less utilized. Feedback highlighted the need for improved usability and additional features.

**Conclusions:**

The study highlights active patient engagement in developing a mobile health app for individuals with rare diseases. Although improvements are necessary for broader acceptance, the app is promising for the management of rare diseases.

**Supplementary information:**

The online version contains supplementary material available at 10.1186/s13023-025-04140-1.

## Background

A disease is defined as rare in the European Union if it affects fewer than 5 in 10,000 individuals [1, 2]. It is estimated that 6,000–8,000 rare diseases (RDs) exist and that 3.5–5.9% of the world’s population is affected. Eighty percent of these diseases are of genetic origin [3]. Consequently, many of these diseases manifest in childhood (50–75%). The low prevalence of RDs presents challenges, including limited expertise, delayed diagnoses, and limited health services. Depping et al. reported that RD patients face deficits in multidisciplinary care, psychological support, and treatment options [4–7]. Considering the use of digital health-associated technologies such as artificial intelligence (AI) or mobile health (mHealth) [8], studies on RDs in these areas are limited [9–12].

In 2002, Istepanian et al. defined mHealth as mobile computing, medical sensors, and communication technologies for healthcare [13–15]. In particular, the development of smartphones and their associated applications have shaped the modus operandi of mHealth [13, 16]. App-based interventions are promising for improving patient outcomes [17]. However, mobile apps for RDs remain underrepresented. Hatem et al. found only 29 apps for various RDs in their 2022 review on the basis of a search of the Google Play Store and Apple Store. In comparison, similar studies reported a higher number of apps for diseases among the top 10 leading causes of death, according to the World Health Organization (WHO) [18]. Examples include ischemic heart diseases (*n* = 38) [19], stroke (*n* = 83) [20], Chronic Obstructive Pulmonary Disease (COPD) (*n* = 13), lower respiratory infections (*n* = 24) [21], neonatal conditions (*n* = 18) [22], lung cancers (*n* = 4) [23], Alzheimer’s disease and dementia (*n* = 17) (US only) [24], diarrheal diseases (*n* = 20) [21], diabetes mellitus (*n* = 120) [25] and kidney disease (*n* = 15) [26], which were identified between 2014 and 2024 in the Google Play Store and Apple Store.

Apps for RDs are tailored for particular diseases or groups of diseases, so they encompass only a small segment of the RD spectrum. Additionally, most of these apps are limited to a few languages, predominantly English. Hatem et al. concluded that a low number of potential users is a poor incentive for the development of an RD app [9].

To address these challenges, the SelEe project (Researching rare diseases in a citizen science approach) developed a mobile device application that enables the self-management of patients with RDs, independent of the type of disease. SelEe is a German citizen science (CS) project involving patients with RDs and their relatives during the entire phase of research [27]. CS is defined as the public’s active involvement in research to expand scientific knowledge, including the design of projects and research questions, collection of data, and result analysis [28, 29]. Accordingly, the research objectives and questions for SelEe were defined by patients, highlighting the need for a customizable app for RDs [27].

CS projects vary in participation levels, from “contributory” (data collection or analysis by citizens) to “collaborative” (refining research questions, analyzing and disseminating data) and “co-creation” (formulating questions and methods) to “citizen-led” (citizens lead all research phases) [30]. Research on the SelEe project has generally fallen into the category of “co-creation”.

To our knowledge, there is no publication available that describes the co-creation process with citizens (in our case patients) in the development of an RD app. Additionally, peer-reviewed literature provides limited evidence of usability testing with the intended user groups for any RD app [9]. Usability is one of the key acceptance criteria for medical applications, especially for mHealth. It ensures that apps are easy to use, intuitive, and meet the needs of both patients and healthcare providers. This is crucial since good usability directly influences user acceptance, engagement, and adherence, which in turn impacts the effectiveness of the app in supporting health outcomes [31]. Without thorough usability evaluation, mHealth apps risk being difficult to use, underutilized, or even misused, ultimately failing to achieve their intended benefits [32]. Therefore, usability should be evaluated not only during the development phase but also after an app has been released [33, 34]. Evaluating usability using methods like user testing, heuristic evaluation, and validated questionnaires, helps identify issues, allowing developers to refine the app’s design and functionality to better fit user needs [35–37].

Consequently, we are the first to present new insights into the co-creation process of an RD app. As part of this process, we evaluated app usage, usability, and functionality after app release in a year-long study.

## Methods

### Co-creation process of the SelEe project

**Fig. 1:**
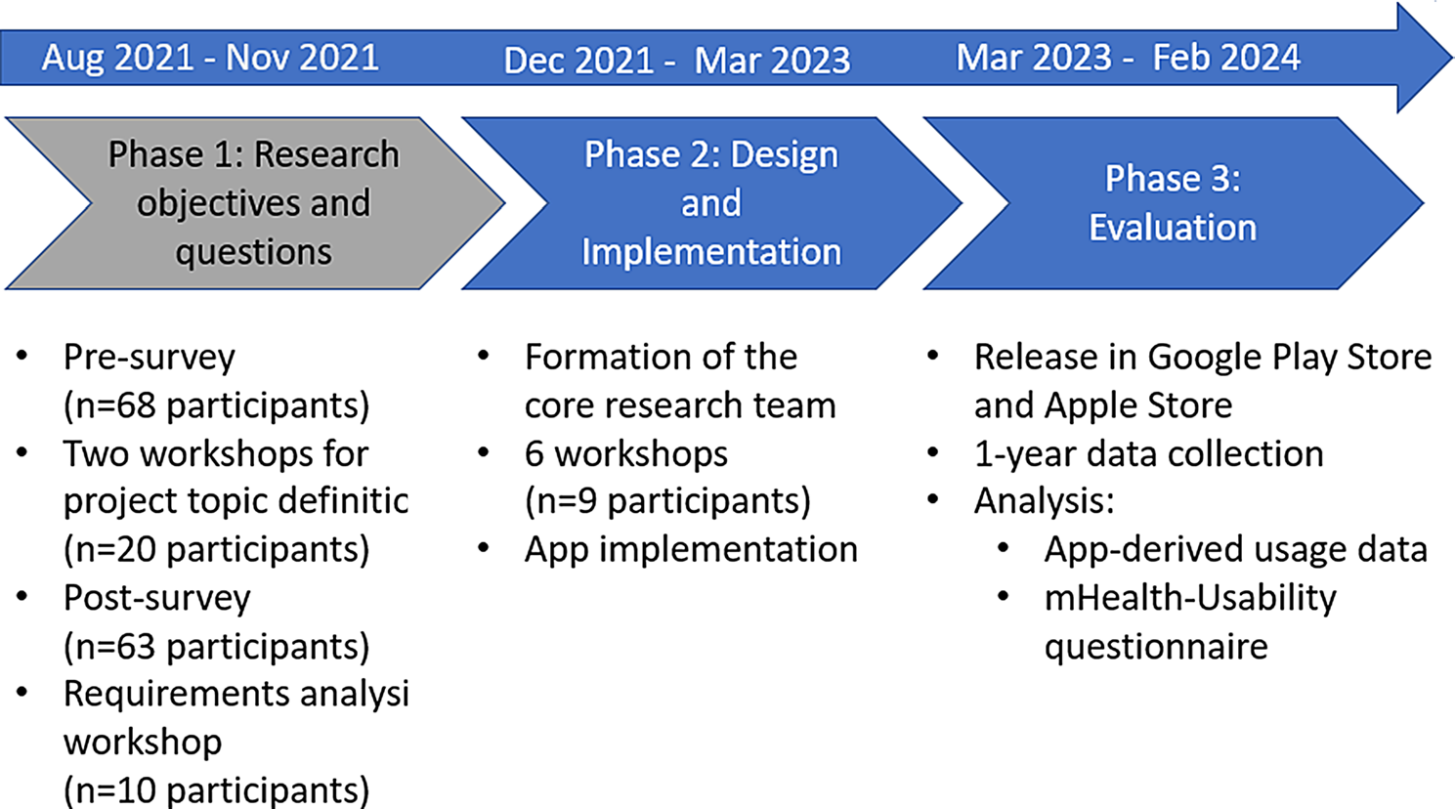
Co-creation process of the SelEe project

The SelEe project consisted of three phases (see Fig. 1). Phase 1 was detailed in a previous publication [27], whereas phases 2 and 3 are reported in this publication.

### Research objective and questions

For phase 1, ethics approval was obtained from the Ethics Committee of the Medical Faculty of Goethe University Frankfurt (reference number: 2021–272). In this phase, patients registered for the project via e-mail in August 2021 (*n* = 68 participants) and suggested topics for the project (pre-survey) [27]. The participants were invited to two online workshops in October and November 2021 to collect ideas for the research objective, and *n* = 20 individuals participated. A follow-up online post-survey determined the final project topic by voting and was distributed to those who completed the pre-survey (*n* = 63 participants). A requirements analysis workshop (*n* = 10 participants) followed to define specific requirements for the app [27]. The main functionality requirements are shown in Table 1. In phase 1, all participants were patients with RDs or relatives.

**Table 1:** Main requirements of the SelEe app

Requirement	Description
Health diary	• Document demographic data, e.g. gender, age, disease • Document custom daily data, e.g. health status, experiences, symptoms, medication • Document recurrent data from doctors’ visits, e.g. findings, doctors’ letter
Visual representation of data	• Visual representation of long-term data trends
Export and printout data	• Export data as a report (for printing), e.g. to show it to physicians or others

### Design and implementation

Based on the insights of phase 1, a core research team was formed (Fig. 1, step 2), inviting all 68 participants to join and participate regularly, with the option to withdraw at any time. Nine individuals (7 of 9 affected by an RD and 2 related persons of an RD affected person) joined the core research team and participated in 6 workshops from December 2021 to September 2022 [38]. Additionally, the team included four professional researchers and one computer science student.

The patient researchers provided suggestions for the app’s functionality and data collection, which were iteratively discussed and adapted. The app was developed by the software team until March 2023 and uses React-Native 0.68 [39] and TypeScript 4, without any patients involvement in programming [40].

For phase 3, another study protocol was submitted to the Ethics Committee of the Medical Faculty of Goethe University Frankfurt (reference number 2022–1039). Users consented to anonymous data storage on a cloud server for research purposes. The app was released in the German language on March 1, 2023, in the Google Play Store and Apple Store.

### Evaluation

#### Setting and sampling

In March 2023, SelEe app users were recruited from the general German population via non-randomized convenience sampling [41]. Recruitment was conducted through social media ads (LinkedIn, Facebook), newspapers, radio stations, and the website of ACHSE (http://www.achse-online.de), which is the head organization of patient organizations for RDs in Germany.

#### Data collection

To evaluate the app, we focused on app usage and usability, collecting data from registered users over a one-year period (March 1, 2023, to February 29, 2024). The evaluation included everyone who downloaded, installed, and created a profile in the app. Users could participate in an in-app survey on usability and functionality (see questionnaire in additional file 1), which was accessible only after profile creation and at least one health diary entry. The survey was divided into three sections. The first part (items 1–5) covered user demographics, reasons for using the app, previous experience with RD-related apps, and how the participants discovered the app. Items 6–21 comprised the mHealth App Usability Questionnaire (MAUQ), a reliable usability questionnaire for mHealth apps that uses a 7-point Likert scale [42]. We used the patient standalone version, covering the subscales “ease of use” (items 6–10), “interface and satisfaction” (items 11–17), and “usefulness” (items 18–21), excluding two items irrelevant to the app in the usefulness subscale [42]. We translated the MAUQ into German through multiple forward and backward translations by independent translators. Two independent, professional translators, whose native language is German and who were familiar with the original English text, translated the MAUQ into German independently of each other. The two German translations were compared and merged into a consolidated version. Inconsistencies were discussed and resolved by consensus. Two other independent translators, whose native language is English and who had no knowledge of the original questionnaire, translated the consolidated German version back into English. Once all steps had been completed, the final German version of the MAUQ was finalized.

The final part (items 22–33) assessed app functionality on a 7-point Likert scale, problem reporting (open-ended questions), and suggestions for improvements (item 34).

#### Data analysis

To analyze the data according to the app usage, descriptive statistics were created: downloads of the SelEe app, created users and profiles, valid profiles, demographics, entered diseases, and usage of the app functionalities. Only profiles with valid diagnoses were included for analysis. A valid entry was defined as either a rare or non-rare disease, a symptom or symptom complex, or an undefined or suspected diagnosis. All other entries were excluded from the analysis (e.g., entries such as “test” or “I’m a doctor”). RDs were classified according to the Orphanet classification, the most common and comprehensive ontology for RDs [43]. Two researchers independently screened the diseases, and any discrepancies were resolved through discussions among all the authors.

For the in-app survey analysis, we calculated descriptive statistics for items 1 to 5. The MAUQ (items 6 to 21) was analyzed as per Zhou et al., who calculated the mean and standard deviation for each subscale and the total usability score [42]. Mean and standard deviation were also calculated for items 22 to 33. Additionally, the open-ended questions were analyzed according to the themes of functionality problems and suggestions for improvement (item 34).

To evaluate the internal consistency of the questionnaire, we calculated Cronbach’s alpha for the parts 2 (MAUQ) and 3 of the questionnaire, considering only Likert scale items related to app usability and functionality. Cronbach’s alpha values of 0.7–0.8 are acceptable, whereas values of approximately 0.9 are ideal [42]. All data analysis steps were performed in Microsoft Excel.

## Results

### SelEe App

The core functionalities of the app are presented in the next subsections (more details in additional file 2).

#### Profile and health diary entries

**Fig. 2:**
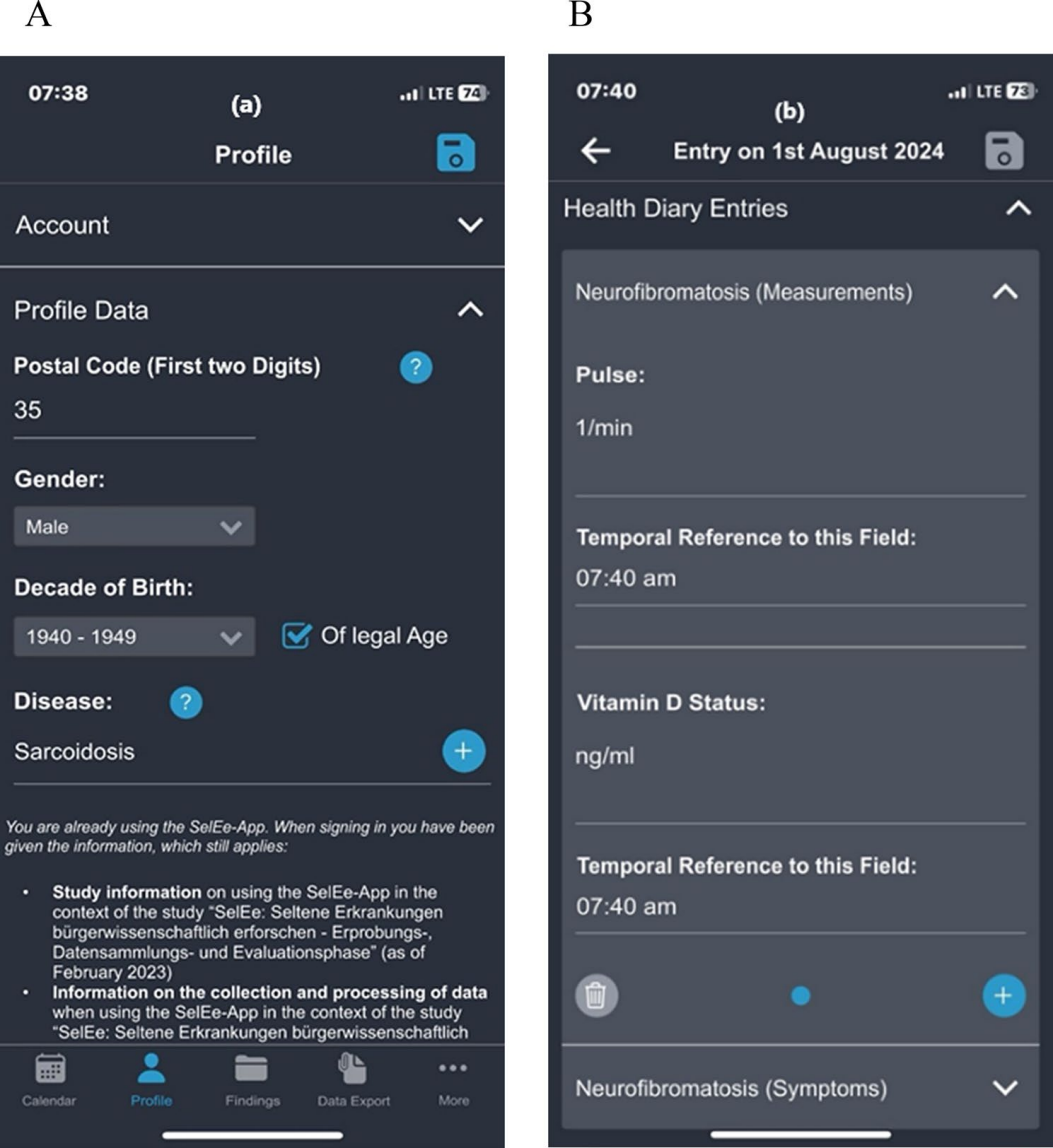
(**A**) Profile view; (**B**) health diary entries in the SelEe app

Demographic data about the user are stored in the profile (see Fig. 2a), which can be edited at any time. Furthermore, options are available, allowing users to consent to study information or notifications.

Health diary entries are split into general and personal sections. The general section includes well-being questions with a slider and optional text. Personal entries (see Fig. 2b), tailored to specific diseases, are user-created or selected from app templates that were created by the core research team. The following app templates are available:

- Vital signs and symptoms: e.g. body weight, fever, and pain
- Circumstances and triggers: Triggering a deterioration in well-being, symptomatology or disease, such as sport, physical exertion, or psychological stress
- Quality of life: General questions on quality of life
- Disease-specific values: Symptoms and measured values for arrhythmogenic heart disease, epilepsy, fatigue-related illnesses, Addison’s disease, Wegener’s granulomatosis, sarcoidosis, and vasculitis

Each health diary entry includes fields storing values in various formats (number, text, date, etc.). Users can fill out entries multiple times per day and edit them for up to 14 days.

#### Findings

**Fig. 3:**
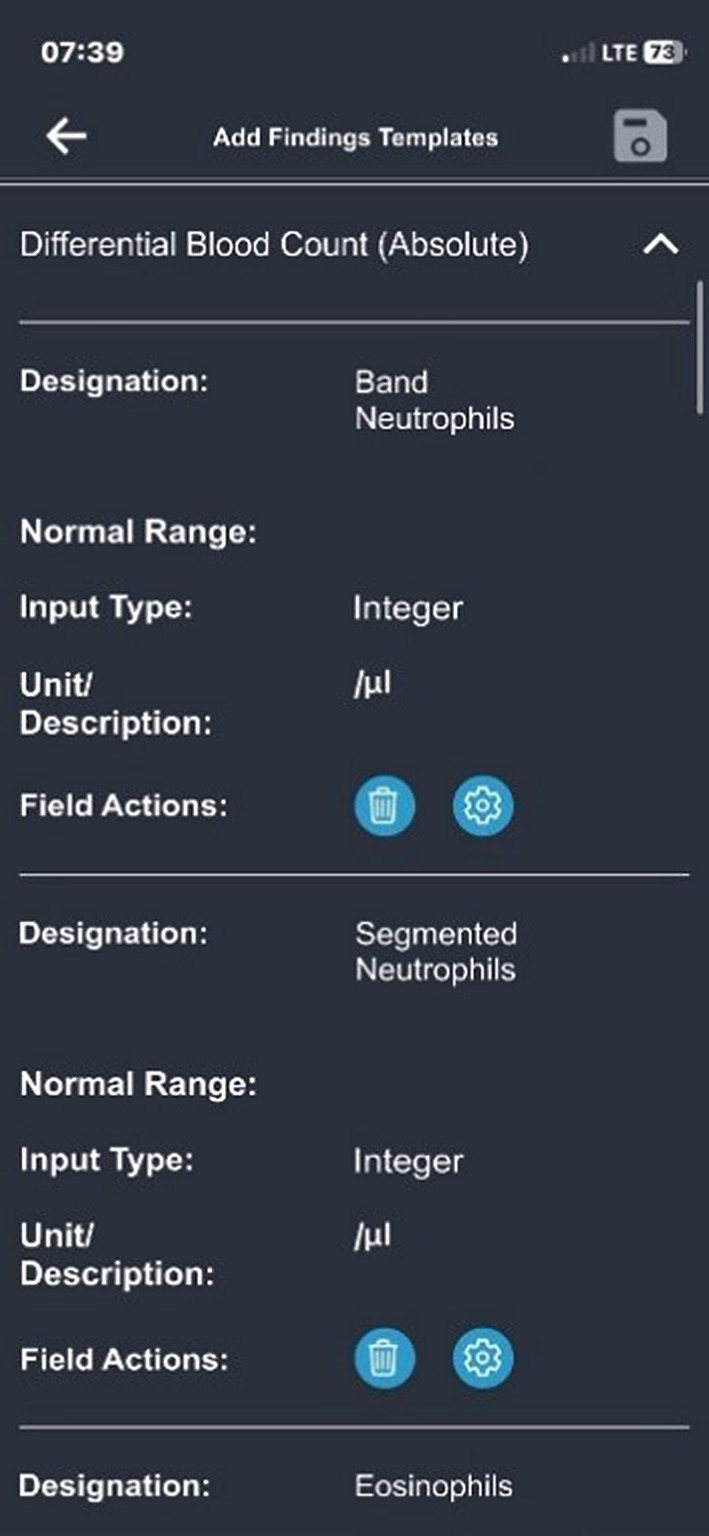
Add findings templates for the differential blood count

The app also offers the option of recording irregular data as findings (e.g., blood values; see Fig. 3). The findings templates are similar to the health diary templates.

A field in a findings template can include a description, input type, standard range, and unit. The following app templates were created together with the core research team:

- Organ involvement: Affected organs
- Arrhythmogenic heart diseases: Echocardiography, magnetic resonance imaging, electrocardiogram, and laboratory results
- Blood differential test (total or relative values): Indicates the cellular composition of white blood cells
- Complete blood count (total or relative values): Includes red/white blood cells, platelets, hemoglobin, and mean corpuscular volume
- Quality of life: Similar to the quality of life in the health diary; this information can alternatively be stored in the findings section.
- Neurofibromatosis: Disease specific findings

#### Export and other functionality

Data can be exported as a PDF with tables and graphs or as a CSV file. The app allows daily or weekly notifications, which are customizable in frequency and time. It is designed to support screen readers, particularly to assist blind users.

### App usage data

**Fig. 4:**
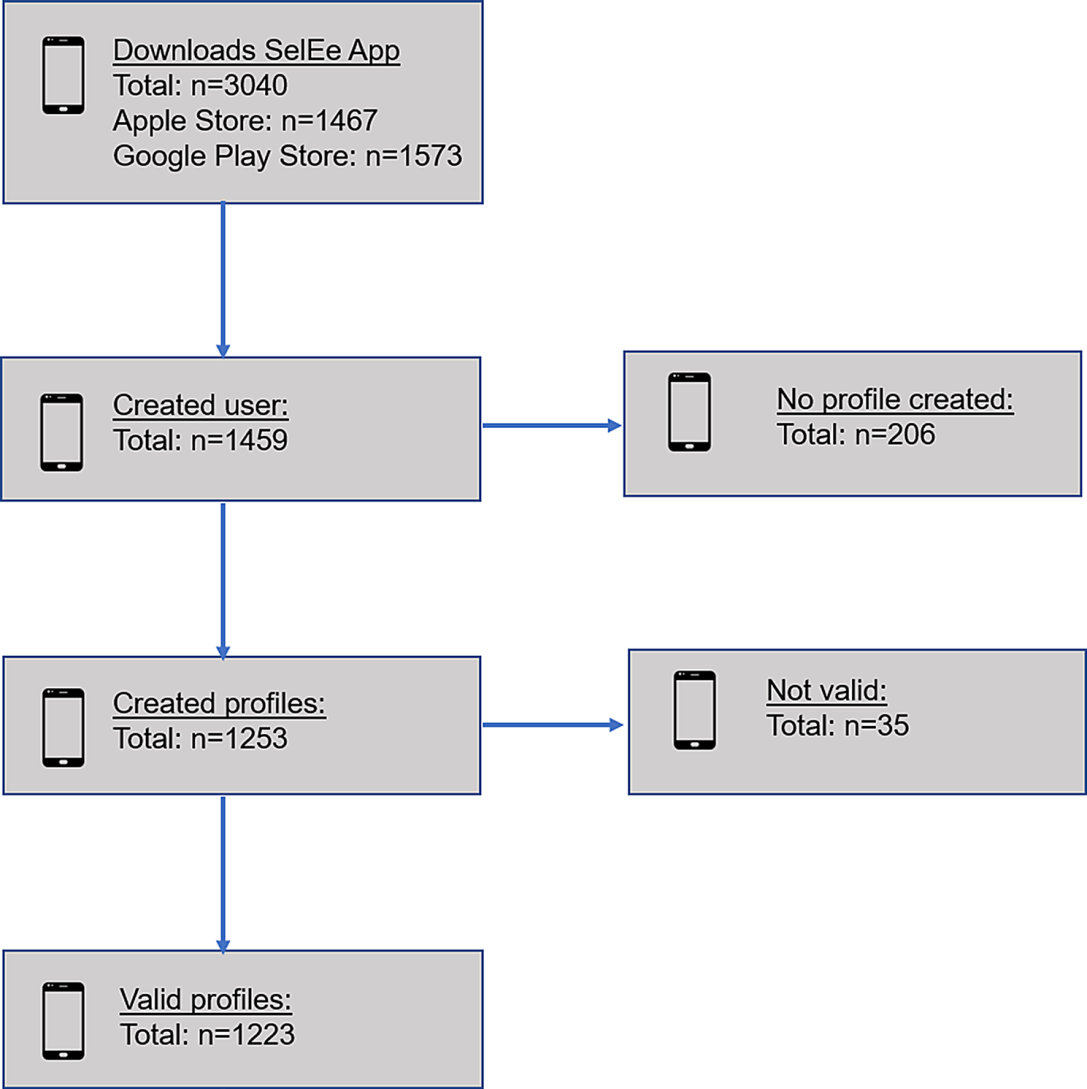
App downloads and registration statistics from the Apple and Google Play Store with valid profiles

A total of 3040 people downloaded the app from the Apple Store (*n* = 1467) and Google Play Store (*n* = 1573) (see Fig. 4). A total of *n* = 1459 persons registered in the app and *n* = 1253 users created a profile. However, *n* = 206 registered users did not create a profile. Finally, after data cleaning, *n* = 1223 valid profiles were available.

#### Demographics

Table 2 shows the demographics of all valid profiles (*n* = 1223). The majority are female (61.4%), males representing 37.4%, and a small proportion identifying as diverse (1.2%). Participants span a wide range of birth decades, with the largest groups born in the 1970s (23.8%) and 1960s (23.0%). Notably, individuals born between 1980 and 1989 make up 15.8% of the sample, while those from the 1990s account for 11.9%.

**Table 2:** Characteristics of the demographics of the participants

Characteristics	Categories	N	%
Gender	Female	751	61.4
	Male	458	37.4
	Diverse	14	1.2
Birth decade (years)	2020–2024	20	1.6
	2010–2019	57	4.7
	2000–2009	69	5.6
	1990–1999	146	11.9
	1980–1989	193	15.8
	1970–1979	290	23.8
	1960–1969	281	23.0
	1950–1959	131	10.7
	1940–1949	32	2.6
	1930–1939	4	0.3

#### Diseases

All valid profile users entered *n* = 1967 diseases, with *n* = 1091 diseases being unique. Table 3 classifies these unique diseases as rare or non-rare and lists the top 10 most frequently entered diseases (*n* = 316).

**Table 3:** Classification of all unique diseases (*n* = 1091) and top 10 diseases (*n* = 316) across participants (*n* = 1223)

Characteristics	Classification	N	%
Unique diseases (*n* = 1091)	Rare	553	50.7
	Not Rare	333	30.5
	Suspected diagnosis/undefined/symptom	205	18.8
Top 10 diseases (*n* = 316)	Addison’s disease	63	20.0
	Ehlers-Danlos syndrome	57	18.0
	Sarcoidosis	32	10.1
	Hashimoto syndrome	31	9.8
	Narcolepsy	28	8.9
	Neurofibromatosis type 1	26	8.2
	Fibromyalgia	24	7.6
	Pituitary insufficiency	19	6.0
	Migraine	18	5.7
	Chronic fatigue syndrome	18	5.7

#### Functionality usage

Of all valid profiles, 432 patients had at least one data entry in the health diary. In total, 6190 entries were made (mean = 14, min = 1, max = 297, SD = 40.3). Furthermore, 983 user templates for health diary entries were created, and 175 findings were entered. Users used the export function 83 times.

**Fig. 5:**
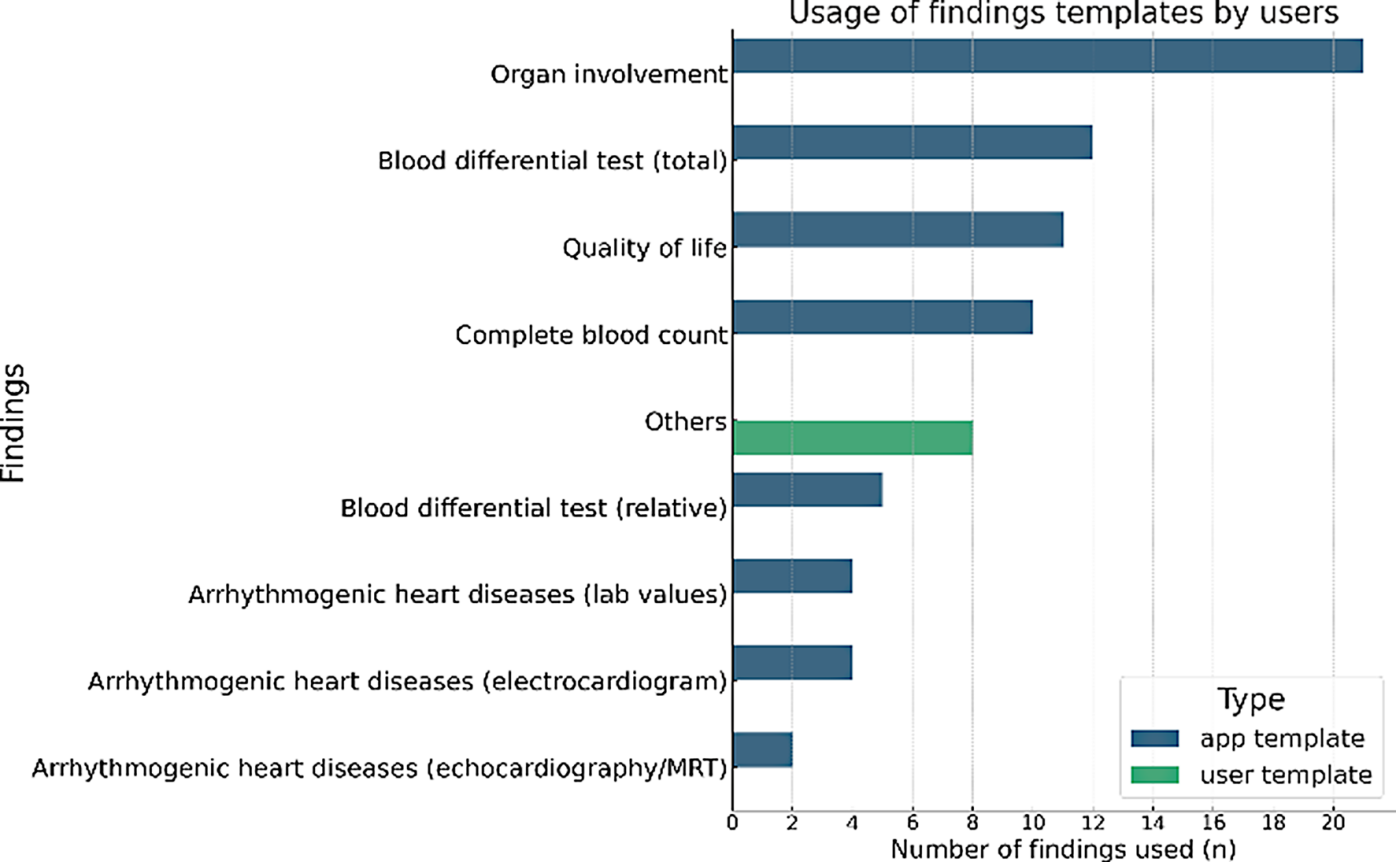
Usage of the findings and their templates across all valid profiles with at least 1 health diary entry (*n* = 432)

Figure 5 shows the usage of findings templates that were available as app templates or user templates. Organ involvement was the most used app template (*n* = 21), whereas user templates were created e.g. for x-ray, edema, alpha-1 disease, lithium in the blood, daily shape and, cardiac contractility modulation (others, *n* = 8).

### Survey results

Of the 432 users who qualified for the in-app survey, 117 (27.08%) successfully filled out the survey. The results of the questionnaire are shown below (details in the additional file 3).

#### Results of questionnaire part 1

Table 4 shows the results of the first part of the questionnaire. The majority (80.4%) use the app because they are affected by a RD, while 11.1% are relatives of someone with a RD, and 6.8% are still seeking a diagnosis. Most respondents are female (67.5%), with males making up 31.6%, and 0.9% identifying as diverse. The largest age group is 18–29 years (48.7%) followed by 30–49 years (30.8%). A significant portion (93.2%) had no prior experience with RD apps. Users mainly discovered the app through advertisements or other sources (50.8%), followed by recommendations from patient organizations (22.9%), private contacts (20.5%), and ACHSE (4.9%).

**Table 4:** Results of the questionnaire part 1 (items 1–5)

Question	Option	N	%
Why do you use the SelEe app?	I am affected by a RD	94	80.4
	I belong to a person with a RD	13	11.1
	I don’t have a diagnosis yet	8	6.8
	Others	2	1.7
Which age group do you belong to?	18–29	14	48.7
	30–49	57	30.8
	50–64	36	12.0
	65 or older	10	8.5
Which gender do you feel you belong to?	Female	79	67.5
	Male	37	31.6
	Diverse	1	0.9
Do you already have previous experience with apps in the field of RDs?	No	109	93.2
	Yes	8	6.8
How did you become aware of the app? (multiple answers possible)	Advertisement or other sources	62	50.8
	Recommendation by a patient organization	28	22.9
	Recommendation from a private contact	25	20.5
	Recommendation by ACHSE	6	4.9
	I am member of the core research team	1	0.8

#### Results of questionnaire part 2

In part 2 of the questionnaire (MAUQ), an overall Cronbach’s alpha of 0.964 was determined. The scores of the subscales “Ease of use”, “Interface and Satisfaction”, and “Usefulness” reached values of 0.937, 0.948, and 0.91, respectively. The results of the participants’ answers for the MAUQ subscales are as follows: “Ease of use” had a mean of 5.54 (SD = 0.12), “Interface and Satisfaction” reached a mean of 5.34 (SD = 0.17), and “Usefulness” had a mean of 4.86 (SD = 0.22). The overall usability across all scales was a mean of 5.19 (SD = 0.29).

For the subscale “Ease of use,” the highest scores were recorded for the items “The navigation was consistent when moving between screens” with a mean of 5.66 (SD = 1.13) and “It was easy for me to learn to use the app” with a mean of 5.60, (SD = 1.25). The lowest score was recorded for the item “Whenever I made a mistake using the app, I could recover easily and quickly”, reaching a mean of 5.37 (SD = 1.27).

For the subscale “Interface and Satisfaction” high scores were obtained for items “I would use this app again” with a mean of 5.57 (SD = 1.31) and “The amount of time involved in using this app has been fitting for me”, mean 5.51 (SD = 1.20). The lowest score was obtained for item “I like the interface of the app”, mean 5.06 (SD = 1.49).

For the “usefulness” subscale, high scores were recorded for the items “The app would be useful for my health and well-being” with a mean of 5.19 (SD = 1.37) and “The app helped me manage my health effectively”, mean 4.83 (SD = 1.41). The lowest score was recorded for the item “This app has all the functions and capabilities I expected it to have”, reaching a mean of 4.68 (SD = 1.43).

#### Results of questionnaire part 3

Table 5 shows the results of items 22–30 (questionnaire part 3, functionality; only Likert scale questions). These items reached a Cronbach’s alpha of 0.84. Overall, users found the app easy to use, with an average score of 5.35 (SD = 1.37). Creating a personal profile was rated the easiest task (Mean = 5.95, SD = 1.19), followed by creating fields for health-data entries (Mean = 5.46, SD = 1.44), and entering health data (Mean = 5.33, SD = 1.33).

**Table 5:** Results of the questionnaire (items 22–33) – functionality (only likert scale items)

Question	Mean	SD
I find creating my own profile to be easy.	5.95	1.19
I find creating fields for the health-data entries in the profile to be easy.	5.46	1.44
I find creating one or more health-data entries to be easy.	5.33	1.33
I find adding a findings-template to be easy.	5.10	1.48
I find creating a finding to be easy.	5.14	1.43
I find using the data export to be easy.	5.12	1.34
*Overall*	5.35	1.37

#### Results of questionnaire part 3 – open-ended questions

The participants identified 18 problems across the app’s functionalities in the open-ended questions. During profile creation, issues included the lack of third-party authentication (e.g., Google), unsaved values, and unclear instructions. For health diary entries, challenges exist in the reliance on instructions to add templates, as well as missing customization options. The overall design was rated as difficult for users without technical knowledge or for older users. Adding findings templates was too complex, with an unintuitive interface and no data import option. Notably, there were no comments regarding data export functionality.

To improve the app, the participants suggested nine enhancements, including automatic health data imported from smart devices. They also recommended improving legibility in dark mode, enabling automatic savings instead of a save icon, and redesigning the app to use a more attractive interface for better usability and clarity.

## Discussion

This publication provides insights into the co-creation process of the CS project SelEe, where we developed the first customizable health app for all RDs. We investigated app usage, usability, and functionality over the course of a year-long study.

### Discussion of results

The app has achieved significant interest, with over 3040 downloads, 1456 registered users, and 1967 entered diseases in one year. The number of different diseases demonstrates a high degree of heterogeneity among users, which is particularly characteristic of RDs [44]. On the MAUQ, the SelEe app achieved a score of 5.19. In comparison, Petracca et al.‘s app for Type A hemophilia scored slightly higher at 5.32 on the MAUQ but it was tested with only 22 participants and not evaluated after the app’s release for the public [45]. However, there is a lack of direct comparative studies with other RD apps. Such comparisons could help better classify the relative effectiveness and user-friendliness of the developed app.

Approximately half of the users who downloaded the SelEe app registered and created profiles. Other studies have shown lower dropout rates after download, such as 23 and 12% [46, 47].

Furthermore, only 432 of the 1223 users who created profiles entered at least one health diary entry. The reasons for this high dropout rate are unclear but may relate to usability issues in creating health diary entries or to consenting to data storage on the cloud server. However, patients with RDs generally have a high willingness to share their data to improve research and healthcare [48]. Further research is needed to determine the reasons for the dropout rate.

Although the app is primarily for RD patients (50.7%), it has also been used by patients without RDs (30.5%) and those with suspected or undefined diagnoses (18.8%), indicating broader interest and need beyond the primary user group. This suggests that the app can record and store reliable health data for various diseases, but further studies are needed to determine measurable positive effects on patients.

Health diary entries are the most frequently used function, indicating that the app is used as intended. However, the findings and export functionalities are used less, likely due to varying relevance for different diseases or usability issues. For instance, the mean scores for the easy use of findings templates (mean 5.10), creating a finding (mean 5.14), and exporting data (mean 5.12) are lower in the questionnaire than those for creating a profile (mean 5.95) and entering health data (mean 5.46). Furthermore, user comments highlight the need for improved design and usability, as intuitive use is limited.

In summary, the app represents a promising, customizable tool for recording and storing health-related data for RDs. The MAUQ indicates a positive ranking for the app’s usefulness for participants’ health and well-being (mean 5.19) and the ability to manage participants’ health effectively (mean 4.86). Despite a good rating for the “ease of use” subscale (mean 5.54), a redesign of the interface is necessary to achieve greater acceptance and use of the app. Furthermore, participants suggested additional functionalities, such as automatic health data import from smart devices or third-party authentication, which should be considered in further development.

### Discussion of methods and limitations

The participants were involved in various project phases, including defining research objectives, project ideas, requirements, development, and app evaluation. This comprehensive methodology can guide future research in developing and evaluating health apps for RDs.

Despite the participants feedback in each workshop, more active design techniques, such as card sorting, as applied by Cho et al., could improve usability of the app [32]. In addition, different designs of the interface could have been tested with A/B tests [49]. Furthermore, the study did not assess the extent of knowledge gained by patients or their satisfaction with the co-creation process. We conducted a survey within the core research team in September 2022 which yielded positive results and we plan to conduct a follow-up study [38].

While the study outlined in this paper evaluated app usage over a year, it is descriptive and exploratory, and no hypothesis testing was performed. As we recruited users who downloaded the app from the app stores, this convenience sampling has some limitations: there is a risk that certain RDs are over- or underrepresented. Especially in RDs with high heterogeneity and differences in prevalence, the sample size is limited [50]. This limits the transferability of the results [51]. Therefore, long-term studies are needed to assess benefits, particularly changes in usage and satisfaction across different age or disease groups and patient outcomes. Furthermore, the in-app survey reported in this paper is limited to users who have actively used the app. Therefore, the results do not reflect individuals who have downloaded the app but did not use it, or who have not made any health diary entries after creating a profile. In addition, not all participants who were able to take part in the in-app survey took part in it. The reason for this cannot be identified from the available data. Nonetheless, the MAUQ appears to be a useful tool for measuring usability and can provide first indications of the usefulness of the app in patient use. One limitation of our self-translated German version of the MAUQ was that the questionnaire had not been validated beforehand. However, the Cronbach’s alpha results are above 0.9 and are therefore similar to those of Tacke et al., who conducted a psychometric analysis to test validity and reliability in their modified German version [52].

Further limitations of the app are its restriction to the German region and language, which limits its applicability, and the lack of an ontology for data entry, which requires manual verification of disease names. By implementing Orphanet classification [38], ICD-10 (German-Modification) [53], or SNOMED-CT [54], disease documentation could be standardized.

## Conclusions

This study offers insights into the co-creation process of the CS project SelEe and underscores the importance of active stakeholder engagement in the development of mHealth applications, especially for RDs. In conclusion, the SelEe app shows promise as a customizable tool for recording and storing health-related data for RDs. While the initial usability and functionality are positive, continued improvements and expanded functionalities are essential for broader acceptance and an enhanced user experience. Future research should focus on long-term studies to assess patient outcomes, especially across different age groups and disease categories.

## Electronic supplementary material

Below is the link to the electronic supplementary material.

Supplementary Material 1

Supplementary Material 2

Supplementary Material 3

## Data Availability

The datasets used and/or analyzed during the current study are available from the corresponding author on reasonable request.

## Abbreviations

ACHSE: Alliance of Chronic Rare Diseases in Germany
AI: Artificial Intelligence
CS: Citizen Science
COPD: Chronic Obstructive Pulmonary Disease
MAUQ: mHealth App Usability Questionnaire
mHealth: Mobile Health
RD: Rare Disease
SelEe: Researching rare diseases in a citizen science approach
WHO: World Health Organization.

## References

[1] European Commission. Rare Diseases. 2024. https://health.ec.europa.eu/non-communicable-diseases/steering-group/rare-diseases. Accessed 20 Sept 2024.

[2] Chung CCY, Chu ATW, Chung BHY. Rare disease emerging as a global public health priority. Front Public Health. 2022;10:1028545. 36339196 10.3389/fpubh.2022.1028545 PMC9632971

[3] Nguengang Wakap S, Lambert DM, Olry A, Rodwell C, Gueydan C, Lanneau V, et al. Estimating cumulative point prevalence of rare diseases: analysis of the Orphanet database. Eur J Hum Genet. 2020;28:165–73. 31527858 10.1038/s41431-019-0508-0 PMC6974615

[4] Dong D, Chung R-N, Chan RHW, Gong S, Xu RH. Why is misdiagnosis more likely among some people with rare diseases than others? Insights from a population-based cross-sectional study in China. Orphanet J Rare Dis. 2020;15:307. 33115515 10.1186/s13023-020-01587-2 PMC7594300

[5] Evans WR, Rafi I. Rare diseases in general practice: recognising the zebras among the horses. Br J Gen Pract. 2016;66:550–51. 27789486 10.3399/bjgp16X687625 PMC5072891

[6] Aymé S, Schmidtke J. Networking for rare diseases: a necessity for Europe. Bundesgesundheitsbl - Gesundheitsforsch - Gesundheitsschutz. 2007;50:1477–83. 10.1007/s00103-007-0381-9 18026888

[7] Depping MK, Uhlenbusch N, von Kodolitsch Y, Klose HFE, Mautner V-F, Löwe B. Supportive care needs of patients with rare chronic diseases: multi-method, cross-sectional study. Orphanet J Rare Dis. 2021;16:44. 33482869 10.1186/s13023-020-01660-w PMC7825171

[8] Mumtaz H, Riaz MH, Wajid H, Saqib M, Zeeshan MH, Khan SE, et al. Current challenges and potential solutions to the use of digital health technologies in evidence generation: a narrative review. Front Digit Health. 2023;5:1203945. 37840685 10.3389/fdgth.2023.1203945 PMC10568450

[9] Hatem S, Long JC, Best S, Fehlberg Z, Nic Giolla Easpaig B, Braithwaite J. Mobile apps for people with rare diseases: review and quality assessment using mobile app rating scale. J Med Internet Res. 2022;24:e36691. 35881435 10.2196/36691 PMC9364167

[10] Schaefer J, Lehne M, Schepers J, Prasser F, Thun S. The use of machine learning in rare diseases: a scoping review. Orphanet J Rare Dis. 2020;15:145. 32517778 10.1186/s13023-020-01424-6 PMC7285453

[11] Schaaf J, Sedlmayr M, Schaefer J, Storf H. Diagnosis of rare diseases: a scoping review of clinical decision support systems. Orphanet J Rare Dis. 2020;15:263. 32972444 10.1186/s13023-020-01536-z PMC7513302

[12] Wojtara M, Rana E, Rahman T, Khanna P, Singh H. Artificial intelligence in rare disease diagnosis and treatment. Clin Transl Sci. 2023;16:2106–11. 37646577 10.1111/cts.13619 PMC10651639

[13] Istepanian RSH. Mobile health (m-health) in retrospect: the known unknowns. Int J Environ Res Public Health. 2022;19:3747. 35409431 10.3390/ijerph19073747 PMC8998037

[14] Istepanian R, Jovanov E, Zhang YT. Introduction to the special section on M-Health: beyond seamless mobility and global wireless health-care connectivity. IEEE Trans Inf Technol Biomed. 2004;8:405–14. 15615031 10.1109/titb.2004.840019

[15] Istepanian RS, Woodward B. M-health fundamentals and applications. 1st. John Wiley & Sons; 2016.

[16] Marcolino MS, Oliveira JAQ, D’Agostino M, Ribeiro AL, Alkmim MBM, Novillo-Ortiz D. The impact of mHealth interventions: systematic review of systematic reviews. JMIR Mhealth Uhealth. 2018;6:e23. 29343463 10.2196/mhealth.8873 PMC5792697

[17] Chong SOK, Pedron S, Abdelmalak N, Laxy M, Stephan A-J. An umbrella review of effectiveness and efficacy trials for app-based health interventions. NPJ Digit Med. 2023;6:233. 38104213 10.1038/s41746-023-00981-x PMC10725431

[18] World Health Organization. The top 10 causes of death. 2024. https://www.who.int/news-room/fact-sheets/detail/the-top-10-causes-of-death. Accessed 20 Sept 2024.

[19] Cruz-Ramos NA, Alor-Hernández G, Colombo-Mendoza LO, Sánchez-Cervantes JL, Rodríguez-Mazahua L, Guarneros-Nolasco LR. mHealth apps for self-management of cardiovascular diseases: a scoping review. Healthcare (Basel). 2022;10:322. 35206936 10.3390/healthcare10020322 PMC8872534

[20] Cao W, Kadir AA, Wang Y, Wang J, Dai B, Zheng Y, et al. Description of apps targeting stroke patients: a review of apps store. Digit Health. 2023;9:20552076231181470. 10.1177/20552076231181473 PMC10278410 37342095

[21] Martínez-Pérez B, de la Torre-Díez I, López-Coronado M, Sainz-De-Abajo B. Comparison of mobile apps for the leading causes of death among different income zones: a review of the literature and app stores. JMIR Mhealth Uhealth. 2014;2:e1. 25099695 10.2196/mhealth.2779 PMC4114467

[22] Richardson B, Dol J, Rutledge K, Monaghan J, Orovec A, Howie K, et al. Evaluation of mobile apps targeted to parents of infants in the neonatal intensive care unit: systematic app review. JMIR Mhealth Uhealth. 2019;7:e11620. 30985282 10.2196/11620 PMC6487340

[23] Charbonneau DH, Hightower S, Katz A, Zhang K, Abrams J, Senft N, et al. Smartphone apps for cancer: a content analysis of the digital health marketplace. Digit Health. 2020;6:2055207620905413. 32110428 10.1177/2055207620905413 PMC7016299

[24] Werner NE, Brown JC, Loganathar P, Holden RJ. Quality of mobile apps for care partners of people with Alzheimer disease and related dementias: mobile app rating scale evaluation. JMIR Mhealth Uhealth. 2022;10:e33863. 35348467 10.2196/33863 PMC9006134

[25] Geirhos A, Stephan M, Wehrle M, Mack C, Messner E-M, Schmitt A, et al. Standardized evaluation of the quality and persuasiveness of mobile health applications for diabetes management. Sci Rep. 2022;12:3639. 35256661 10.1038/s41598-022-07544-2 PMC8901695

[26] Lewis RA, Lunney M, Chong C, Tonelli M. Identifying mobile applications aimed at self-management in people with chronic kidney disease. Can J Kidney Health Dis. 2019;6:2054358119834283. 30899533 10.1177/2054358119834283 PMC6419251

[27] Neff M, Storf H, Vasseur J, Scheidt J, Zerr T, Khouri A, et al. Identifying project topics and requirements in a citizen science project in rare diseases: a participative study. Orphanet J Rare Dis. 2022;17:357. 36104743 10.1186/s13023-022-02514-3 PMC9476337

[28] Trejo M, Canfield I, Robinson JO, Guerrini CJ. How biomedical citizen scientists define what they do: it’s all in the name. AJOB Empir Bioeth. 2021;12:63–70. 32990526 10.1080/23294515.2020.1825139 PMC8021393

[29] Rowbotham S, McKinnon M, Leach J, Lamberts R, Hawe P. Does citizen science have the capacity to transform population health science? Crit Public Health. 2019;29:118–28.

[30] Marks L, Laird Y, Trevena H, Smith BJ, Rowbotham S. A scoping review of citizen science approaches in chronic disease prevention. Front Public Health. 2022;10. 10.3389/fpubh.2022.743348 PMC9125037 35615030

[31] Anders C, Moorthy P, Svensson L, Müller J, Heinze O, Knaup P, et al. Usability and user experience of an mHealth app for therapy support of patients with breast cancer: mixed methods study using eye tracking. JMIR Hum Factors. 2024;11:e50926. 10.2196/50926 PMC10951836 38441959

[32] Cho H, Yen PY, Dowding D, Merrill JA, Schnall R. A multi-level usability evaluation of mobile health applications: a case study. J Biomed Inf. 2018;86:79–89. 10.1016/j.jbi.2018.08.012 PMC6448568 30145317

[33] Deniz-Garcia A, Fabelo H, Rodriguez-Almeida AJ, et al. Quality, usability, and effectiveness of mHealth apps and the role of artificial intelligence: current scenario and challenges. J Med Internet Res. 2023;25:e44030. 37140973 10.2196/44030 PMC10196903

[34] Wang Q, Liu J, Zhou L, Tian J, Chen X, Zhang W, et al. Usability evaluation of mHealth apps for elderly individuals: a scoping review. BMC Med Inf Decis Mak. 2022;22:317. 10.1186/s12911-022-02064-5 PMC9717549 36461017

[35] Klasnja P, Hartzler A, Powell C, Pratt W. Supporting cancer patients’ unanchored health information management with mobile technology. AMIA Annu Symp Proc. 2011;732–41. PMC3243297 22195130

[36] Specht L, Scheible R, Boeker M, Farin-Glattacker E, Kampel N, Schmölz M, et al. Evaluating the acceptance and usability of an Independent, noncommercial search engine for medical information: cross-sectional questionnaire study and user behavior tracking analysis. JMIR Hum Factors. 2025;23:e56941. 10.2196/56941 PMC11803324 39847765

[37] Shen Y, Wang S, Shen Y, Tan S, Dong Y, Qin W, Zhuang Y. Evaluating the usability of mHealth apps: an evaluation model based on task analysis methods and eye movement data. Healthcare (Basel). 2024;12:1310. 38998845 10.3390/healthcare12131310 PMC11241497

[38] Schaaf J, Khouri A, Zerr T, Scheidt J, Neff M, Storf H. Rare diseases in citizen science - preliminary experiences in developing a personal health app. Stud Health Technol Inf. 2024;310:1151–55. 10.3233/SHTI231145 38269995

[39] Sciandra L, Shakow A. Announcing react native 0.68. 2022. https://reactnative.dev/blog/2022/03/30/version-068. Accessed 20 Sept 2024.

[40] TypeScript M. 2020. https://www.typescriptlang.org/. Accessed 20 Sept 2024.

[41] Andrade C. The inconvenient truth about convenience and purposive samples. Indian J Psychol Med. 2021;43:86–88. 34349313 10.1177/0253717620977000 PMC8295573

[42] Zhou L, Bao J, Setiawan IMA, Saptono A, Parmanto B. The mHealth app usability questionnaire (MAUQ): development and validation study. JMIR Mhealth Uhealth. 2019;7. 10.2196/11500 PMC6482399 30973342

[43] Weinreich SS, Mangon R, Sikkens JJ, Teeuw ME, Cornel MC. Orphanet: a European database for rare diseases. Ned Tijdschr Geneeskd. 2008;152:518–19. 18389888

[44] Whicher D, Philbin S, Aronson N. An overview of the impact of rare disease characteristics on research methodology. Orphanet J Rare Dis. 2018;13:14. 29351763 10.1186/s13023-017-0755-5 PMC5775563

[45] Petracca F, Tempre R, Cucciniello M, Ciani O, Pompeo E, Sannino L, et al. An electronic patient-reported outcome mobile app for data collection in type a Hemophilia: design and usability study. JMIR Form Res. 2021;5:e25071. 34855619 10.2196/25071 PMC8686465

[46] Goyal S, Morita PP, Picton P, Seto E, Zbib A, Cafazzo JA. Uptake of a consumer-focused mHealth application for the assessment and prevention of heart disease: the <30 days study. JMIR Mhealth Uhealth. 2016;4:e32. 27012937 10.2196/mhealth.4730 PMC4824871

[47] Mallafré-Larrosa M, Papi G, Trilla A, Ritchie D. Development and promotion of an mHealth app for adolescents based on the European code against cancer: retrospective cohort study. JMIR Cancer. 2023;9:e48040. 38015612 10.2196/48040 PMC10716759

[48] Courbier S, Dimond R, Bros-Facer V. Share and protect our health data: an evidence-based approach to rare disease patients’ perspectives on data sharing and data protection - quantitative survey and recommendations. Orphanet J Rare Dis. 2019;14:175. 31300010 10.1186/s13023-019-1123-4 PMC6625078

[49] Strecker P, Boeker M, Buechner S, Scheible R. Usability evaluation of a modern multilingual MeSH browser. Stud Health Technol Inf. 2022;295:37–40. 10.3233/SHTI220653 35773799

[50] Bietz MJ, Bloss CS, Calvert S, Godino JG, Gregory J, Claffey MP, et al. Opportunities and challenges in the use of personal health data for health research. J Am Med Inf Assoc. 2016;23:e42–8. 10.1093/jamia/ocv118 PMC4954630 26335984

[51] Kidwell KM, Roychoudhury S, Wendelberger B, Scott J, Moroz T, Yin S, et al. Application of Bayesian methods to accelerate rare disease drug development: scopes and hurdles. Orphanet J Rare Dis. 2022;17:186. 35526036 10.1186/s13023-022-02342-5 PMC9077995

[52] Tacke T, Nohl-Deryk P, Lingwal N, Reimer LM, Starnecker F, Güthlin C, et al. The German version of the mHealth app usability questionnaire (GER-MAUQ): translation and validation study in patients with cardiovascular disease. Digit Health. 2024;10:20552076231225170. 10.1177/20552076231225168 PMC10832428 38303970

[53] Bundesinstitut für Arzneimittel und Medizinprodukte. Internationale statistische Klassifikation der Krankheiten und verwandter Gesundheitsprobleme 10. Revision German modification version. 2024. 2024. https://klassifikationen.bfarm.de/icd-10-gm/kode-suche/htmlgm2024/index.htm. Accessed 20 Sep 2024.

[54] Gaudet-Blavignac C, Foufi V, Bjelogrlic M, Lovis C. Use of the systematized nomenclature of medicine clinical terms (SNOMED CT) for processing free text in health care: systematic scoping review. J Med Internet Res. 2021;23:e24594. 33496673 10.2196/24594 PMC7872838

