# Supplementary material for: Co-creation process of an app for people with rare diseases - a citizen science approach
Source: Orphanet J Rare Dis. 2025 Nov 27;20:614. doi: 10.1186/s13023-025-04140-1 (PMC12659587; doi:10.1186/s13023-025-04140-1)
Supplement: Supplementary file 1 — Supplementary Material 1 [file 13023_2025_4140_MOESM1_ESM.pdf]

Additional file 1 – Questionnaire

Part 1: Usage profile

Item No.	Question (German)	Question (English)	Answer option (German)	Answer option (English)
1	Aus welchem Grund nutzen Sie die SelEe-App?	Why do you use the SelEe app?	Ich bin betroffen von einer Seltenen Erkrankung	I am affected by a rare disease
			Ich bin Angehöriger einer Person mit einer Seltenen Erkrankung	I belong to a person with a rare disease
			Ich habe bisher keine Diagnose	I don’t have a diagnosis yet
			Sonstiges	Others
2	Welcher Altersgruppe sind Sie zugehörig?	Which age group do you belong to?	18-29	18-29
			30-49	30-49
			50-64	50-64
			65 oder älter	65 or older
3	Welchem Geschlecht fühlen Sie sich zugehörig?	Which gender do you feel you belong to?	Weiblich	Female
			Männlich	Male
			Divers	Diverse
4	Haben Sie bereits Vorerfahrungen mit Apps im Bereich Seltene Erkrankungen?	Do you already have previous experience with apps in the field of rare diseases?	Nein	No
			Ja	Yes
5	Wie sind Sie auf die App aufmerksam geworden?	How did you become aware of the app?	Werbung oder andere Quellen	Advertisement or other sources
			Empfehlung durch eine Patientenorganisation	Recommendation by a patient organization
			Empfehlung durch einen privaten Kontakt	Recommendation from a private contact
			Empfehlung durch ACHSE	Recommendation by ACHSE
			Ich bin Mitglied des Kernforschungsteams	I am member of the core research team

Part 2: mHealth App Usability Questionnaire (MAUQ)

Item No.	Question (German)	English (English)	Answer option (German)	Answer option (English)
6	Die App war einfach zu bedienen.	The app was easy to use.	• Stimme überhaupt nicht zu<br>• Stimme nicht zu<br>• Stimme eher nicht zu<br>• Stimme weder zu noch lehne ich ab<br>• Stimme eher zu<br>• Stimme voll und ganz zu	• Strongly disagree<br>• Disagree<br>• Somewhat disagree<br>• Neither agree or disagree<br>• Somewhat agree<br>• Agree<br>• Strongly agree
7	Es war einfach für mich, die Nutzung der App zu erlernen.	It was easy for me to learn to use the app.	• Stimme überhaupt nicht zu<br>• Stimme nicht zu<br>• Stimme eher nicht zu<br>• Stimme weder zu noch lehne ich ab<br>• Stimme eher zu<br>• Stimme voll und ganz zu	• Strongly disagree<br>• Disagree<br>• Somewhat disagree<br>• Neither agree or disagree<br>• Somewhat agree<br>• Agree<br>• Strongly agree
8	Die Navigation war beim Wechsel zwischen den einzelnen Seiten der App einheitlich.	The navigation was consistent when moving between screens.	• Stimme überhaupt nicht zu<br>• Stimme nicht zu<br>• Stimme eher nicht zu<br>• Stimme weder zu noch lehne ich ab<br>• Stimme eher zu<br>• Stimme voll und ganz zu	• Strongly disagree<br>• Disagree<br>• Somewhat disagree<br>• Neither agree or disagree<br>• Somewhat agree<br>• Agree<br>• Strongly agree
9	Die Nutzeroberfläche der App ermöglichte es mir, alle von der App angebotenen Funktionen zu nutzen.	The interface of the app allowed me to use all the functions.	• Stimme überhaupt nicht zu<br>• Stimme nicht zu<br>• Stimme eher nicht zu<br>• Stimme weder zu noch lehne ich ab<br>• Stimme eher zu<br>• Stimme voll und ganz zu	• Strongly disagree<br>• Disagree<br>• Somewhat disagree<br>• Neither agree or disagree<br>• Somewhat agree<br>• Agree<br>• Strongly agree
10	Wenn mir bei der Verwendung der App ein Fehler unterlief, konnte ich ihn leicht und schnell beheben.	Whenever I made a mistake using the app, I could recover easily and quickly.	• Stimme überhaupt nicht zu<br>• Stimme nicht zu<br>• Stimme eher nicht zu	• Strongly disagree<br>• Disagree<br>• Somewhat disagree

Item No.	Question (German)	English (English)	Answer option (German)	Answer option (English)
			• Stimme weder zu noch lehne ich ab<br>• Stimme eher zu<br>• Stimme voll und ganz zu	• Neither agree or disagree<br>• Somewhat agree<br>• Agree<br>• Strongly agree
11	Mir gefällt die Nutzeroberfläche der App.	I like the interface of the app.	• Stimme überhaupt nicht zu<br>• Stimme nicht zu<br>• Stimme eher nicht zu<br>• Stimme weder zu noch lehne ich ab<br>• Stimme eher zu<br>• Stimme voll und ganz zu	• Strongly disagree<br>• Disagree<br>• Somewhat disagree<br>• Neither agree or disagree<br>• Somewhat agree<br>• Agree<br>• Strongly agree
12	Die Informationen in der App waren gut organisiert, so dass ich Informationen, die ich benötigte, leicht finden konnte.	The information in the app was well organised, so I could easily find the information I needed.	• Stimme überhaupt nicht zu<br>• Stimme nicht zu<br>• Stimme eher nicht zu<br>• Stimme weder zu noch lehne ich ab<br>• Stimme eher zu<br>• Stimme voll und ganz zu	• Strongly disagree<br>• Disagree<br>• Somewhat disagree<br>• Neither agree or disagree<br>• Somewhat agree<br>• Agree<br>• Strongly agree
13	Die App hat den Fortschritt meiner Aktion angemessen angezeigt und mich darüber informiert.	The app adequately acknowledged and provided information to let me know the progress of my action.	• Stimme überhaupt nicht zu<br>• Stimme nicht zu<br>• Stimme eher nicht zu<br>• Stimme weder zu noch lehne ich ab<br>• Stimme eher zu<br>• Stimme voll und ganz zu	• Strongly disagree<br>• Disagree<br>• Somewhat disagree<br>• Neither agree or disagree<br>• Somewhat agree<br>• Agree<br>• Strongly agree
14	Ich fühle mich wohl, wenn ich diese App in einer sozialen Umgebung verwende.	I feel comfortable using this app in social settings.	• Stimme überhaupt nicht zu<br>• Stimme nicht zu<br>• Stimme eher nicht zu<br>• Stimme weder zu noch lehne ich ab<br>• Stimme eher zu<br>• Stimme voll und ganz zu	• Strongly disagree<br>• Disagree<br>• Somewhat disagree<br>• Neither agree or disagree<br>• Somewhat agree<br>• Agree<br>• Strongly agree
15	Der Zeitaufwand für die Nutzung dieser App war für mich angemessen.	The amount of time involved in using this app has been fitting for me.	• Stimme überhaupt nicht zu<br>• Stimme nicht zu	• Strongly disagree<br>• Disagree

Item No.	Question (German)	English (English)	Answer option (German)	Answer option (English)
			• Stimme eher nicht zu<br>• Stimme weder zu noch lehne ich ab<br>• Stimme eher zu<br>• Stimme voll und ganz zu	• Somewhat disagree<br>• Neither agree or disagree<br>• Somewhat agree<br>• Agree<br>• Strongly agree
16	Ich würde diese App wiederverwenden.	I would use this app again.	• Stimme überhaupt nicht zu<br>• Stimme nicht zu<br>• Stimme eher nicht zu<br>• Stimme weder zu noch lehne ich ab<br>• Stimme eher zu<br>• Stimme voll und ganz zu	• Strongly disagree<br>• Disagree<br>• Somewhat disagree<br>• Neither agree or disagree<br>• Somewhat agree<br>• Agree<br>• Strongly agree
17	Insgesamt bin ich mit dieser App zufrieden.	Overall, I am satisfied with this app.	• Stimme überhaupt nicht zu<br>• Stimme nicht zu<br>• Stimme eher nicht zu<br>• Stimme weder zu noch lehne ich ab<br>• Stimme eher zu<br>• Stimme voll und ganz zu	• Strongly disagree<br>• Disagree<br>• Somewhat disagree<br>• Neither agree or disagree<br>• Somewhat agree<br>• Agree<br>• Strongly agree
18	Die App wäre nützlich für meine Gesundheit und mein Wohlbefinden.	The app would be useful for my health and well-being.	• Stimme überhaupt nicht zu<br>• Stimme nicht zu<br>• Stimme eher nicht zu<br>• Stimme weder zu noch lehne ich ab<br>• Stimme eher zu<br>• Stimme voll und ganz zu	• Strongly disagree<br>• Disagree<br>• Somewhat disagree<br>• Neither agree or disagree<br>• Somewhat agree<br>• Agree<br>• Strongly agree
19	Die App hat mir geholfen, meine Gesundheit effektiv zu verwalten.	The app helped me manage my health effectively.	• Stimme überhaupt nicht zu<br>• Stimme nicht zu<br>• Stimme eher nicht zu<br>• Stimme weder zu noch lehne ich ab<br>• Stimme eher zu<br>• Stimme voll und ganz zu	• Strongly disagree<br>• Disagree<br>• Somewhat disagree<br>• Neither agree or disagree<br>• Somewhat agree<br>• Agree<br>• Strongly agree

Item No.	Question (German)	English (English)	Answer option (German)	Answer option (English)
20	Diese App hat alle Funktionen und Möglichkeiten, die ich erwartet habe	This app has all the functions and capabilities I expected it to have.	• Stimme überhaupt nicht zu<br>• Stimme nicht zu<br>• Stimme eher nicht zu<br>• Stimme weder zu noch lehne ich ab<br>• Stimme eher zu<br>• Stimme voll und ganz zu	• Strongly disagree<br>• Disagree<br>• Somewhat disagree<br>• Neither agree or disagree<br>• Somewhat agree<br>• Agree<br>• Strongly agree
21	Ich konnte die App auch dann nutzen, wenn die Internetverbindung schlecht oder nicht verfügbar war.	I could use the app even when the Internet connection was poor or not available.	• Stimme überhaupt nicht zu<br>• Stimme nicht zu<br>• Stimme eher nicht zu<br>• Stimme weder zu noch lehne ich ab<br>• Stimme eher zu<br>• Stimme voll und ganz zu	• Strongly disagree<br>• Disagree<br>• Somewhat disagree<br>• Neither agree or disagree<br>• Somewhat agree<br>• Agree<br>• Strongly agree

Scale: 1 - strongly disagree, 2 - disagree, 3 - somewhat disagree, 4 - neither agree nor disagree, 5 - somewhat agree, 6 - agree, 7 - strongly agree

Part 3: Functionality

Item No.	Question (German)	Question (English)	Answer option (German)	Answer option (English)
22	Das Anlegen eines eigenen Profils empfinde ich als einfach.	I find creating my own profile to be easy.	• Stimme überhaupt nicht zu<br>• Stimme nicht zu<br>• Stimme eher nicht zu<br>• Stimme weder zu noch lehne ich ab<br>• Stimme eher zu<br>• Stimme voll und ganz zu	• Strongly disagree<br>• Disagree<br>• Somewhat disagree<br>• Neither agree or disagree<br>• Somewhat agree<br>• Agree<br>• Strongly agree
24	Das Anlegen eigener Felder im Profil empfinde ich als einfach.	I find creating fields for the health-data entries in the profile to be easy.	• Stimme überhaupt nicht zu<br>• Stimme nicht zu<br>• Stimme eher nicht zu<br>• Stimme weder zu noch lehne ich ab<br>• Stimme eher zu<br>• Stimme voll und ganz zu	• Strongly disagree<br>• Disagree<br>• Somewhat disagree<br>• Neither agree or disagree<br>• Somewhat agree<br>• Agree<br>• Strongly agree
26	Das Anlegen ein oder mehrerer Kalendereinträge empfinde ich als einfach.	I find creating one or more health-data entries to be easy.	• Stimme überhaupt nicht zu<br>• Stimme nicht zu<br>• Stimme eher nicht zu<br>• Stimme weder zu noch lehne ich ab<br>• Stimme eher zu<br>• Stimme voll und ganz zu	• Strongly disagree<br>• Disagree<br>• Somewhat disagree<br>• Neither agree or disagree<br>• Somewhat agree<br>• Agree<br>• Strongly agree
28	Das Hinzufügen einer Befundvorlage empfinde ich als einfach.	I find adding a findings-template to be easy.	• Stimme überhaupt nicht zu<br>• Stimme nicht zu<br>• Stimme eher nicht zu<br>• Stimme weder zu noch lehne ich ab<br>• Stimme eher zu<br>• Stimme voll und ganz zu	• Strongly disagree<br>• Disagree<br>• Somewhat disagree<br>• Neither agree or disagree<br>• Somewhat agree<br>• Agree<br>• Strongly agree
30	Das Anlegen eines Befundes empfinde ich als einfach.	I find creating a finding to be easy.	• Stimme überhaupt nicht zu<br>• Stimme nicht zu<br>• Stimme eher nicht zu<br>• Stimme weder zu noch lehne ich ab<br>• Stimme eher zu<br>• Stimme voll und ganz zu	• Strongly disagree<br>• Disagree<br>• Somewhat disagree<br>• Neither agree or disagree<br>• Somewhat agree<br>• Agree<br>• Strongly agree

32	Die Nutzung des Datenexports empfinde ich als einfach.	I find using the data export to be easy.	• Stimme überhaupt nicht zu<br>• Stimme nicht zu<br>• Stimme eher nicht zu<br>• Stimme weder zu noch lehne ich ab<br>• Stimme eher zu<br>• Stimme voll und ganz zu	• Strongly disagree<br>• Disagree<br>• Somewhat disagree<br>• Neither agree or disagree<br>• Somewhat agree<br>• Agree<br>• Strongly agree

Scale: 1 - strongly disagree, 2 - disagree, 3 - somewhat disagree, 4 - neither agree nor disagree, 5 - somewhat agree, 6 - agree, 7 - strongly agree

Item No	Question (German)	Question (English)	Answer option (German)	Answer option (English)
23	Beim Anlegen eines eigenen Profils hatte ich folgende Probleme:	I had the following problems when creating my own profile:	Freitext	Free text
25	Beim Anlegen der eigenen Felder hatte ich folgende Probleme:	I had the following problems when creating my own fields:	Freitext	Free text
27	Beim Anlegen ein oder mehrerer Kalendereinträge hatte ich folgende Probleme:	I had the following problems when creating one or more health-diary entries:	Freitext	Free text
29	Beim Hinzufügen einer Befundvorlage hatte ich folgende Probleme:	I had the following problems when adding a findings template:	Freitext	Free text
31	Beim Anlegen eines Befundes hatte ich folgende Probleme:	I had the following problems when creating a finding:	Freitext	Free text
33	Bei der Nutzung des Exports hatte ich die folgenden Probleme:	I had the following problems when using the data export:	Freitext	Free text
34	Welche weiteren Verbesserungsvorschläge haben Sie bezüglich der App?	What other suggestions do you have for improving the app?	Freitext	Free text
