# Supplementary material for: Co-creation process of an app for people with rare diseases - a citizen science approach
Source: Orphanet J Rare Dis. 2025 Nov 27;20:614. doi: 10.1186/s13023-025-04140-1 (PMC12659587; doi:10.1186/s13023-025-04140-1)

# Additional file 2 – Description of the SelEe App

The following document describes the basic functions of the SelEe App:

- Profile
- Findings and Findings Templates
- Health Diary and Health Diary Templates
- Data Export

The example screenshots of the SelEe App shown in this document have been translated into English for the purpose of publication.

## Profile

After registering on the SelEe app, users can access the profile menu to manage account settings, enter profile information, and update data options. Demographic data, including postcode, gender, birth decade, and one or more health conditions, are stored, as illustrated in the following figures.

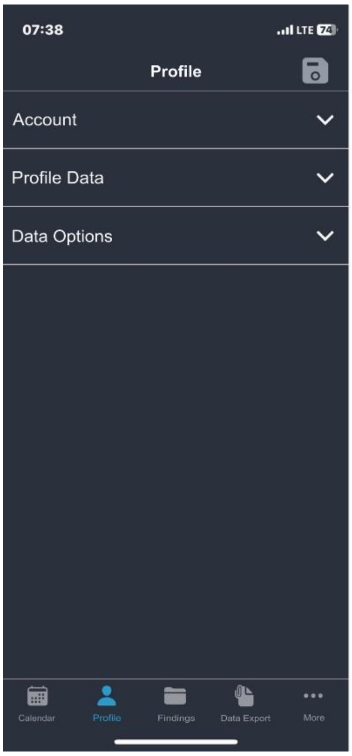


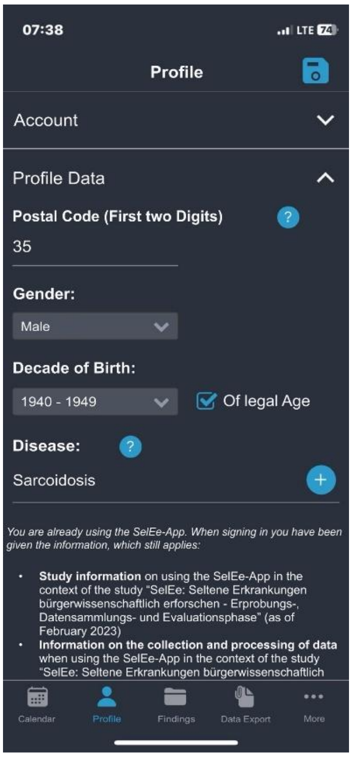


Additionally, users must provide consent to the privacy policy for the anonymized storage of data and for participating in the survey (optional). You can also choose whether to receive email or push notifications, such as reminders for the health diary or new findings. Under the 'Data Option' section, users can add health diary templates. It is also possible to customize whether the general section of the health diary, which includes questions about physical and mental health, should be displayed.

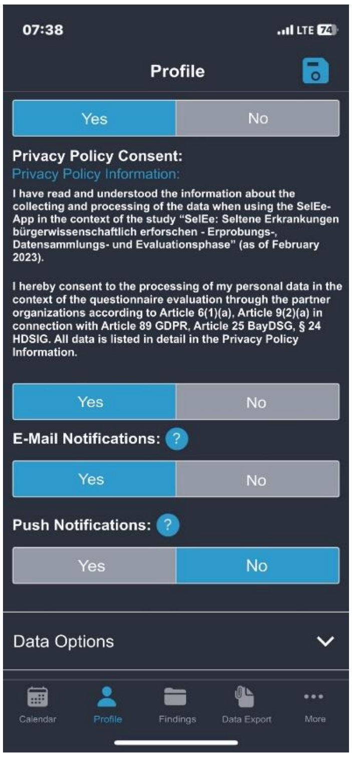


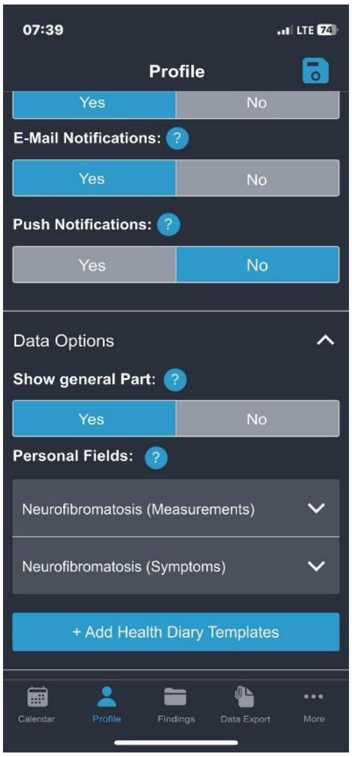


## Health Diary and Health Diary Templates

The SelEe app offers a health diary feature that allows users to track various health-related data. These diary entries can be either user-generated or selected from app-templates designed by the project's core research team. As illustrated in the following figures, users can choose from app-provided templates (e.g., General (Symptoms)) or create their own by specifying a custom name (e.g., My Disease).

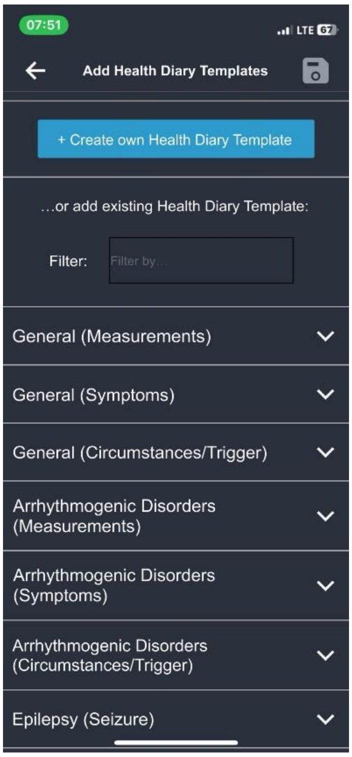


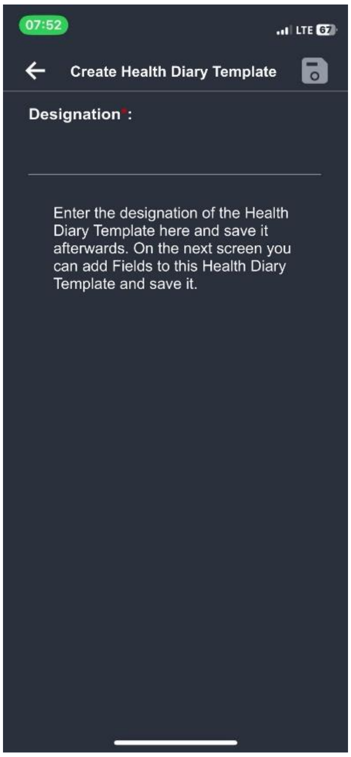

Each Health Diary Template must include at least one field. Every field has a specific designation, indicating whether it is mandatory, the type of input required (such as text or number), and whether a temporal reference is needed (e.g., recording the time of a symptom or event).

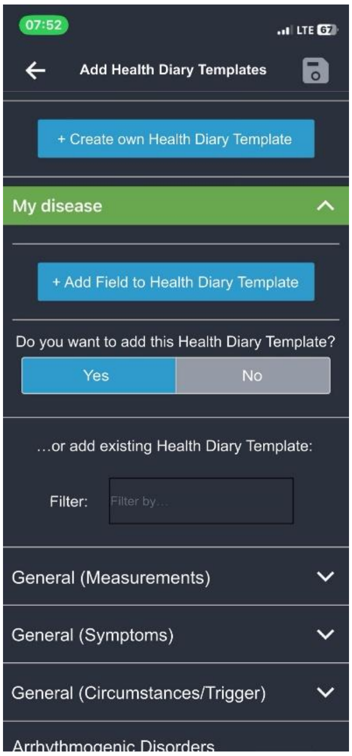


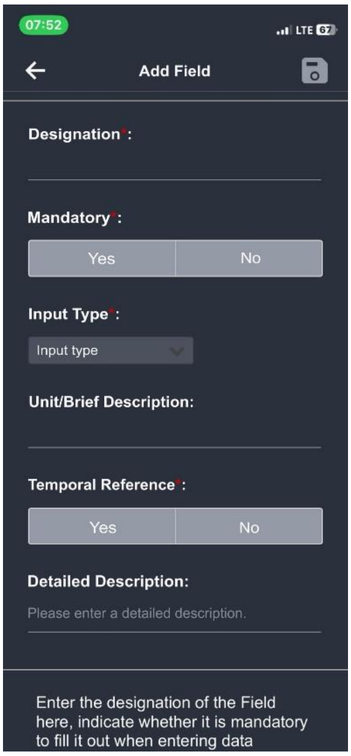


After a health diary template is successfully configured, the diary becomes ready for use. Users can document entries for up to 14 days in the past, but future entries, are not allowed. When a calendar day is selected, a section with general questions will appear if this option is set in the profile. Documentation is then completed in the diary using the predefined templates.

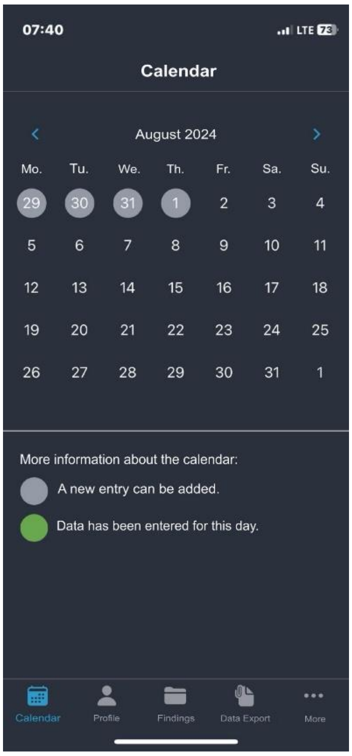


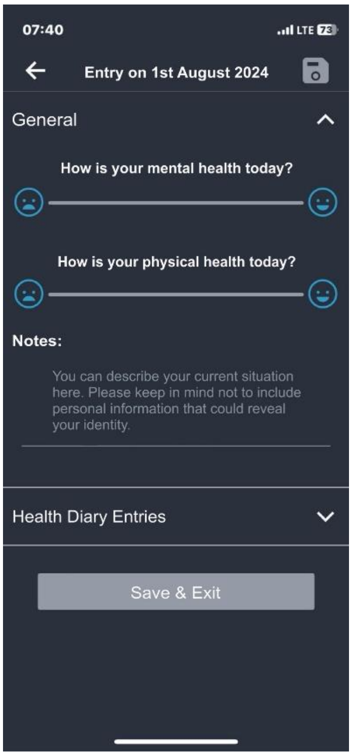


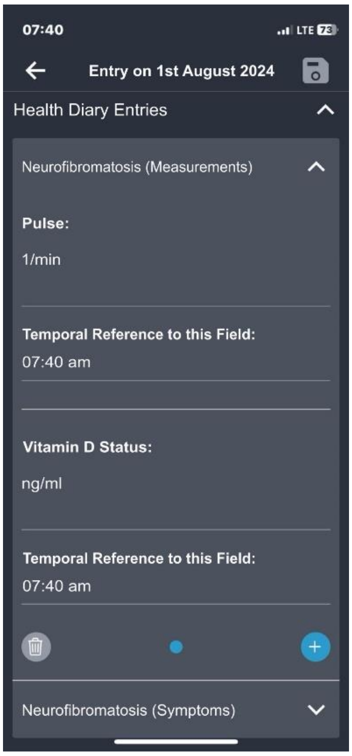

## Findings and Findings Templates

The app also provides the option to record irregular data, such as laboratory values, as findings. The findings templates function similarly to the health diary templates. By clicking the "Create Own Finding Template" button, users can generate a custom template.

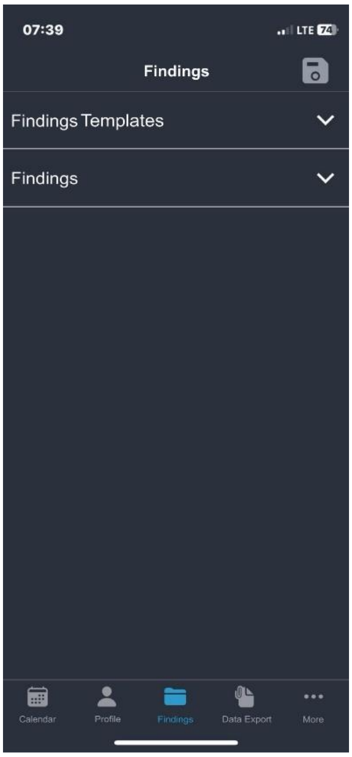


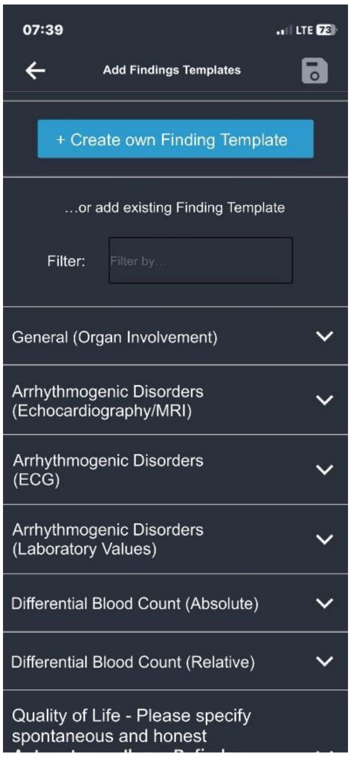


A field in a findings template can have a designation, input type, normal range (e.g. for laboratory values) and unit. Furthermore, a detailed description is optional. Findings can also be used without predefined templates, but a field must be created in this step.

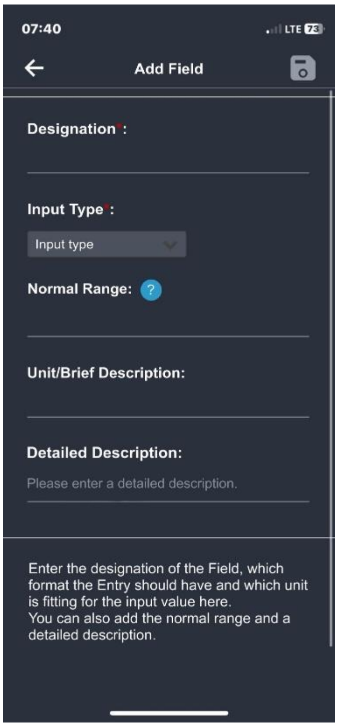


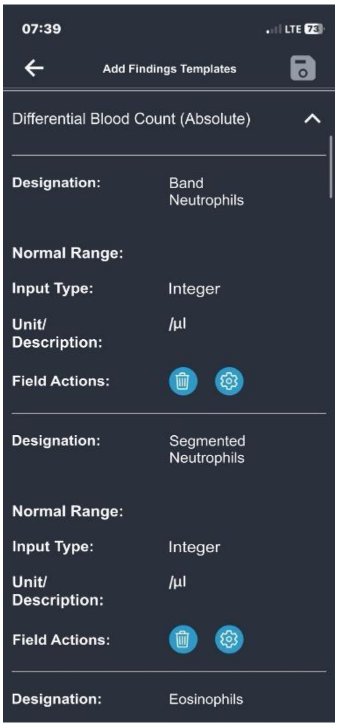


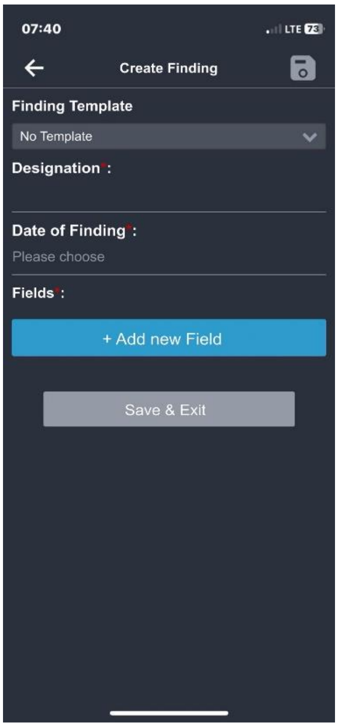

# Data Export

Data entered in the SelEe app can be exported in .csv or .pdf format. To do this, users must specify a start and end date for the health diary entries. Selecting the "Calendar" checkbox provides an overview of all entries within the chosen period. The "Single Diagrams" option allows users to create trend diagrams for specific values, such as physical or mental health. The "Combined Diagrams" option enables the display of multiple fields in a single diagram.

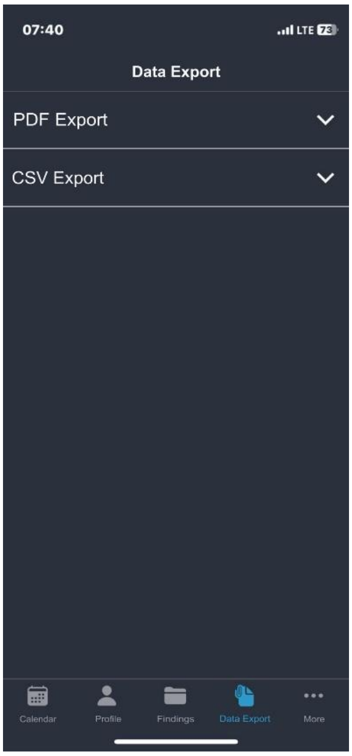


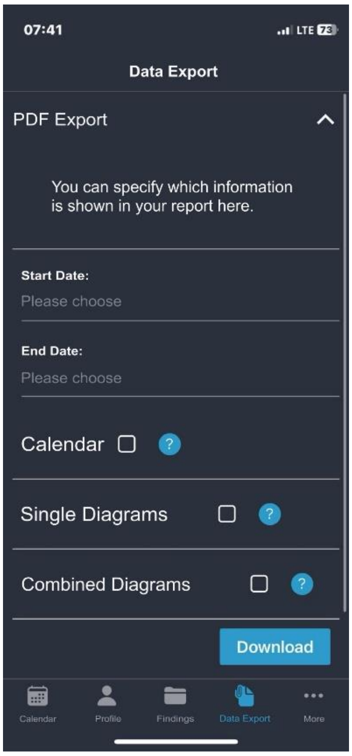


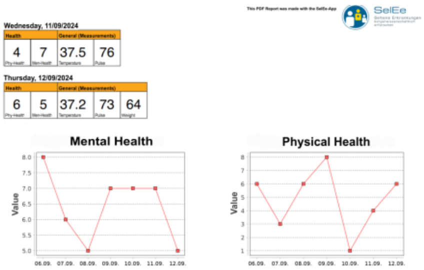

Supplement: Supplementary file 2 — Supplementary Material 2 [file 13023_2025_4140_MOESM2_ESM.pdf]
