# Supplementary material for: Co-creation process of an app for people with rare diseases - a citizen science approach
Source: Orphanet J Rare Dis. 2025 Nov 27;20:614. doi: 10.1186/s13023-025-04140-1 (PMC12659587; doi:10.1186/s13023-025-04140-1)
Supplement: Supplementary file 3 — Supplementary Material 3 [file 13023_2025_4140_MOESM3_ESM.pdf]

Additional file 3 – Results of the questionnaire

Part 1: Usage profile

Item No.	Question (German)	Question (English)	Answer option (English)	N	%
1	Aus welchem Grund nutzen Sie die SelEe-App?	Why do you use the SelEe app?	I am affected by a rare disease	94	80.4
			I belong to a person with a rare disease	13	11.1
			I don't have a diagnosis yet	8	6.8
			Others	2	1.7
2	Welcher Altersgruppe sind Sie zugehörig?	Which age group do you belong to?	18-29	14	48.7
			30-49	57	30.8
			50-64	36	12.0
			65 or older	10	8.5
3	Welchem Geschlecht fühlen Sie sich zugehörig?	Which gender do you feel you belong to?	Female	79	67.5
			Male	37	31.6
			Diverse	1	0.9
4	Haben Sie bereits Vorerfahrungen mit Apps im Bereich Seltene Erkrankungen?	Do you already have previous experience with apps in the field of rare diseases?	No	109	93.2
			Yes	8	6.8
5	Wie sind Sie auf die App aufmerksam geworden?	How did you become aware of the app?	Advertisement or other sources	62	50.8
			Recommendation by a patient organization	28	22.9
			Recommendation from a private contact	25	20.5
			Recommendation by ACHSE	6	4.9
			I am member of the core research team	1	0.8

Part 2: mHealth App Usability Questionnaire (MAUQ)

Cronbach alpha = 0.964, 16 items

Item No.	Question (German)	English (English)	Mean (SD)
**Ease of Use** (Cronbach alpha = 0.937, 5 items)			
6	Die App war einfach zu bedienen	The app was easy to use.	5.46 (1.34)
7	Es war einfach für mich, die Nutzung der App zu erlernen	It was easy for me to learn to use the app.	5.60 (1.25)
8	Die Navigation war beim Wechsel zwischen den einzelnen Seiten der App einheitlich.	The navigation was consistent when moving between screens.	5.66 (1.13)
9	Die Nutzeroberfläche der App ermöglichte es mir, alle von der App angebotenen Funktionen zu nutzen.	The interface of the app allowed me to use all the functions.	5.60 (1.14)
10	Wenn mir bei der Verwendung der App ein Fehler unterlief, konnte ich ihn leicht und schnell beheben.	Whenever I made a mistake using the app, I could recover easily and quickly.	5.37 (1.27)
Total subscale			5.54 (0.12)

Item No.	Question (German)	English (English)	Mean (SD)
**Interface and satisfaction** (Cronbach alpha = 0.948, 7 items)			
11	Mir gefällt die Nutzeroberfläche der App.	I like the interface of the app.	5.06 (1.49)
12	Die Informationen in der App waren gut organisiert, so dass ich Informationen, die ich benötigte, leicht finden konnte.	The information in the app was well organized, so I could easily find the information I needed.	5.32 (1.40)
13	Die App hat den Fortschritt meiner Aktion angemessen angezeigt und mich darüber informiert.	The app adequately acknowledged and provided information to let me know the progress of my action.	5.18 (1.35)
14	Ich fühle mich wohl, wenn ich diese App in einer sozialen Umgebung verwende.	I feel comfortable using this app in social settings.	5.35 (1.23)
15	Der Zeitaufwand für die Nutzung dieser App war für mich angemessen.	The amount of time involved in using this app has been fitting for me.	5.51 (1.20)
16	Ich würde diese App wiederverwenden.	I would use this app again.	5.57 (1.31)
17	Insgesamt bin ich mit dieser App zufrieden.	Overall, I am satisfied with this app.	5.37 (1.35)
Total subscale			5.34 (0.17)
**Usefulness** (Cronbach alpha = 0.91, 4 items)			
18	Die App wäre nützlich für meine Gesundheit und mein Wohlbefinden.	The app would be useful for my health and well-being.	5.19 (1.37)
19	Die App hat mir geholfen, meine Gesundheit effektiv zu verwalten.	The app helped me manage my health effectively.	4.83 (1.41)
20	Diese App hat alle Funktionen und Möglichkeiten, die ich erwartet habe.	This app has all the functions and capabilities I expected it to have.	4.68 (1.43)
21	Ich konnte die App auch dann nutzen, wenn die Internetverbindung schlecht oder nicht verfügbar war.	I could use the app even when the Internet connection was poor or not available.	4.75 (1.17)
Total subscale			4.86 (0.22)
**Overall scale**			5.19 (0.29)

Scale: 1 - strongly disagree, 2 - disagree, 3 - somewhat disagree, 4 - neither agree nor disagree, 5 - somewhat agree, 6 - agree, 7 - strongly agree

Part 3: Functionality

(Cronbach alpha = 0.84, items 22, 24, 26, 28, 30, 32)

Item No.	Question (German)	Question (English)	Mean (SD)
22	Das Anlegen eines eigenen Profils empfinde ich als einfach.	I find creating my own profile to be easy.	5.95 (1.19)
24	Das Anlegen eigener Felder im Profil empfinde ich als einfach.	I find creating fields for the health-data entries in the profile to be easy.	5.46 (1.44)
26	Das Anlegen ein oder mehrerer Kalendereinträge empfinde ich als einfach.	I find creating one or more health-data entries to be easy.	5.33 (1.33)
28	Das Hinzufügen einer Befundvorlage empfinde ich als einfach.	I find adding a findings-template to be easy.	5.10 (1.48)
30	Das Anlegen eines Befundes empfinde ich als einfach.	I find creating a finding to be easy.	5.14 (1.43)
32	Die Nutzung des Datenexports empfinde ich als einfach.	I find using the data export to be easy.	5.12 (1.34)

Scale: 1 - strongly disagree, 2 - disagree, 3 - somewhat disagree, 4 - neither agree nor disagree, 5 - somewhat agree, 6 - agree, 7 - strongly agree

Item No	Question (German)	Question (English)	Responses (German)	Responses (translated to English)
23	Beim Anlegen eines eigenen Profils hatte ich folgende Probleme:	I had the following problems when creating my own profile:	“Leider keine Unterstützung von z.B. Google Login.”	“Unfortunately, no support of e.g. Google login.”
			“Die Felder wurden nicht gespeichert.”	“The fields have not been saved."
			“Die Seiten sind unübersichtlich.”	“The pages are confusing.”
			“Zu viele Optionen zum Ausfüllen, es dauert mir zu lange.”	“Too many options to fill in, it takes too long for me.”
			“Ich bin nicht sicher, ob das Profil korrekt gespeichert wurde.”	“I'm not sure if the profile was saved correctly.”
25	Beim Anlegen der eigenen Felder hatte ich folgende Probleme:	I had the following problems when creating my own fields:	“Ich konnte wiegen einmal wöchentlich nicht so eintragen, musste täglich eintragen.”	"I couldn't enter my weight once a week, I had to enter it daily."
			“Ich musste die Anleitung schauen um zu verstehen wie ich einen Block zufüge. Der Filter sollte auch Blockeinträge filtern und nicht nur Blockkategorien. Der Filter stand jetzt ist nutzlos, da es eh alphabetisch sortiert ist und nicht so viele Einträge gibt.”	“I had to look at the instructions to understand how to add a block. The filter should also filter block entries and not just block categories. The filter as it stands now is useless as it is sorted alphabetically anyway and there are not that many entries.”
			“Mir fehlt ein Wenn/Dann Funktion. Bei einigen Feldern benötige ich die zeitliche Komponente nur bei einem zuvor ausgewähltem Ja. Für die Ernährung wäre ein übergreifendes Feld mit Unterfeldern zu den Mahlzeiten oder Tageszeiten sinnvoll.”	“I am missing an if/then function. For some fields, I only need the time component for a previously selected Yes. For nutrition, an overarching field with subfields for meals or times of day would be useful.”
			“Das ewige Scrollen nervt, die Felder sind viel zu groß (Tablet) und es gibt keine Eingabehilfen Stemming. Außerdem werden explizit angelegte Blöcke und Felder immer geschlossen angezeigt und man muss sie manuell aufklappen. Ohne meine umfangreichen langjährigen Computerkenntnisse wäre dir App kaum zu verstehen. Das UX Design ist einfach mies und stümperhaft, eben als ob's der HiWi Praktikant gemacht hat.”	“The constant scrolling is annoying, the fields are far too large (tablet) and there are no input aids . In addition, explicitly created blocks and fields are always displayed closed and you have to open them manually. Without my many years of extensive computer knowledge, the app would be almost impossible to understand. The UX design is simply lousy and bumbling, as if it was done by a student.”
			„Wenn man das Grundprinzip entschlüsselt hat, ist es einfach. Aber bis dahin habe ich etwas gebraucht.“	“Once you've deciphered the basic principle, it's easy. But it took me a while to get there.”
27	Beim Anlegen ein oder mehrerer Kalendereinträge hatte ich folgende Probleme:	I had the following problems when creating one or more health-diary entries:	“Ich habe den Sinn der Kalender Einträge noch nicht ganz verstanden.”	“I haven't quite understood the meaning of the health-diary entries yet."
			„Seit 3 Tagen kann ich nicht mehr speichern.”	“I have not been able to save for 3 days.”
			“Ein deutliches Abwählen (ausgrauen o.ä.) wäre übersichtlich.”	“A clear deselection (gray out or similar) would be clear."

Item No	Question (German)	Question (English)	Responses (German)	Responses (translated to English)
			“Die Kalendertage anzuklicken ist nicht einfach. Vor allem für ältere Leute gibt es hier Schwierigkeiten. Da es die eine Funktion ist, die man täglich nutzt, sollten die Tage größer sein. Und wieso kann ich die Daten von vor 2 Wochen nicht zufügen? Was, wenn ich mir vor 2 Wochen hab Blut abnehmen lassen und jetzt erst die Ergebnisse bekomme.”	"Clicking on the calendar days is not easy. There are difficulties here, especially for older people. As this is the one function that you use every day, the days should be larger. And why can't I add the dates from 2 weeks ago? What if I had my blood taken two weeks ago and am only now getting the results?"
29	Beim Hinzufügen einer Befundvorlage hatte ich folgende Probleme:	I had the following problems when adding a findings template:	“Zu kompliziert.”	“Too complicated.”
			“Ich habe keine Ahnung wie das funktionieren soll.”	“I don’t know how it works.”
			“Leider gar nicht intuitiv und verständlich. Es scheint auch keine Möglichkeit zum Import zu geben? Also z. B Fotografieren eines bestehenden Befundes mit automatischer Übernahme der Werte.”	“Unfortunately not at all intuitive and understandable. There also doesn't seem to be an option for importing? For example, photographing an existing finding with automatic transfer of the values."
31	Beim Anlegen eines Befundes hatte ich folgende Probleme:	I had the following problems when creating a finding:	“Ich wollte eine Datei anhängen, damit ich alles an einem Ort verwalten kann.”	"I wanted to attach a file so that I could manage everything in one place."
33	Bei der Nutzung des Exports hatte ich die folgenden Probleme:	I had the following problems when using the data export:	No answers	No answers
34	Welche weiteren Verbesserungsvorschläge haben Sie bezüglich der App?	What other suggestions do you have for improving the app?	“Bitte unbedingt eine API, um Gesundheitsdaten von Smartwatches und anderen Trackern automatisiert oder zumindest auf Knopfdruck zu importieren.”	“An API to import health data from smartwatches and other trackers automatically or at least at the touch of a button.”
			“App sollte auf mehreren Geräten gleichzeitig laufen, falls man selbst keinen Eintrag machen kann.”	App should run on several devices at the same time if you cannot make an entry yourself.
			“Schönere Oberfläche.”	“More beautiful interface.”
			“Befunde einscannen.”	“Scan findings.”
			“Auflistung der Erkrankung und Auswahl dieser. Anschließend gezielt auf diese Erkrankung eingehen.”	“List and select the disease. Then specifically address this disease.”
			“Im Darkmode ist die blaue Schrift auf g rauem Hintergrund kaum zu lesen! Wies o gibt es kein onboarding zur Profilerstel lung, bevor man in der App ist. Das Popu p mit bitte erstellen sie ein Profil zeigt ei nfach faule Entwicklung, oder schlechte Entscheidungen bei der Usability. Das S peichern-Etikett Symbol ist aus den 90er Jahren und total seltsam. Das, dass nicht automatisch gespeichert wird ist keine gu te usability. Zumindest beim popup könn	In dark mode, the blue font on a gray background is barely legible! Why is there no onboarding to create a profile before you are in the app? The popup with please create a profile just shows lazy development, or bad usability decisions. The save label icon is from the 90s and totally weird. The fact that it is not saved automatically is no good usability. At least the popup could have an additional option like save & exit. In

Item No	Question (German)	Question (English)	Responses (German)	Responses (translated to English)
			te man eine zusätzliche Option haben wie speichern & verlassen. Zudem sollte das Verlassen rot eingefärbt sein, damit der Nutzer weiß, dass Daten nicht gespeicher t werden. Komplettes Neudesign. Furcht bare Nutzererfahrung."	addition, the exit should be colored red so that the user knows that data is not saved. Complete redesign. Terrible user experience."
			"Mehr Übersichtlichkeit."	"More clarity".
			"Noch mehr vereinfachen."	"Simplify even more."
			"Ich würde mich freuen, wenn die App einfacher erklärt wird."	"I would be happy if the app was explained more simply."
